# Plasma branched-chain and aromatic amino acids correlate with the gut microbiota and severity of Parkinson’s disease

**DOI:** 10.1038/s41531-022-00312-z

**Published:** 2022-04-21

**Authors:** Yi Zhang, Xiaoqin He, Yiwei Qian, Shaoqing Xu, Chengjun Mo, Zheng Yan, Xiaodong Yang, Qin Xiao

**Affiliations:** 1 Department of Neurology and Institute of Neurology, Ruijin Hospital, Shanghai Jiao Tong University School of Medicine, Shanghai, China; 2 Shanghai Institute of Nutrition and Health, Chinese Academy of Sciences, Shanghai, China

**Keywords:** Parkinson's disease, Neurodegeneration

## Abstract

Disturbances of circulating amino acids have been demonstrated in patients with Parkinson’s disease (PD). However, there have been no consistent results for branched-chain amino acids (BCAAs) and aromatic amino acids (AAAs), and related factors have not been explored. We aimed to explore plasma BCAA and AAA profiles in PD patients, and identify their correlations with clinical characteristics and the gut microbiota. Plasma BCAA (leucine, isoleucine, and valine) and AAA (tyrosine and phenylalanine) levels were measured in 106 PD patients and 114 controls. Fecal samples were collected from PD patients for microbiota sequencing and functional analysis. We found that plasma BCAAs and tyrosine were decreased in PD patients. BCAAs and AAAs were correlated with clinical characteristics and microbial taxa, and, in particular, they were negatively correlated with the Hoehn and Yahr stage. Compared with early PD patients, BCAA and AAA levels were even lower, and microbial composition was altered in advanced PD patients. Predictive functional analysis indicated that predicted genes numbers involved in BCAA biosynthesis were lower in advanced PD patients. What’s more, the fecal abundances of critical genes (*ilvB*, *ilvC*, *ilvD*, and *ilvN*) involved in BCAA biosynthesis were reduced and fecal BCAA concentrations were lower in advanced PD patients. In conclusion, the disturbances of plasma BCAAs and AAAs in PD patients may be related to the gut microbiota and exacerbated with PD severity. The microbial amino acid metabolism may serve as a potential mechanistic link.

## Introduction

Parkinson’s disease (PD) is the second most common neurodegenerative disorder, affecting about 1% of people over 60 years of age^1^. The main neuropathological characteristics of PD are loss of dopaminergic neurons in substantia nigra and intracellular accumulation of alpha-synuclein aggregates (Lewy bodies)^2^. Disturbances in plasma metabolites, including amino acids, fatty acids, and bile acids, have been demonstrated in PD patients based on a metabolomics approach^3^. The plasma amino acid levels (e.g., alanine and arginine) have been reported to correlate with disease duration and medications in PD^4^. Furthermore, previous studies have investigated the alterations of some amino acids in blood samples to identify potential biomarkers to assist in the diagnosis of PD^5,6^. The signature of serum amino acids differs in patients with early and advanced PD, suggesting that specific amino acid profiles could serve as a potential biomarker of PD severity^4^.

Branched-chain amino acids (BCAAs), which refer to leucine (Leu), isoleucine (Ile), and valine (Val), are essential amino acids and serve as nitrogen donors in the brain to maintain the glutamate-glutamine cycle between astrocytes and neurons^7^. BCAAs could promote the catabolism of glutamate by activating ﻿glutamate dehydrogenase^8^. Transamination of BCAAs generates branched-chain ketoacids, which contribute to the “buffering effect” on potentially toxic levels of glutamate^7^. Increasing glutamate has been reported to be closely related to the occurrence and development of PD through its excitotoxicity, oxidative stress, and immunoexcitotoxicity^9^. Thus, BCAAs may exert beneficial effects in PD via modifying glutamate metabolism. Tyrosine (Tyr) and phenylalanine (Phe), which are aromatic amino acids (AAAs), are critical substrates for the production of dopamine, the deficient neurotransmitter in PD. Although there have been several studies on the alteration of circulating BCAAs and AAAs in patients with PD^5,10–13^, the results have not been consistent, and correlations with clinical characteristics have not yet been investigated. Furthermore, potential factors associated with this alteration need to be explored.

The gut microbiota has been recognized as a key regulator of health and disease, and can influence the absorption and metabolism of ingested nutrients, with potentially profound effects on host physiology^14^. Notably, the gut microbiota can promote the production and utilization of amino acids^15^, which can be absorbed across the gut and accumulate in the blood^16^. Thus, the gut microbiota could influence plasma amino acid levels. There is growing evidence that PD patients suffer from gut microbiota dysbiosis^17–20^, suggesting that the gut microbiota is involved in the pathogenesis of PD. However, whether the disturbances in plasma amino acids are associated with the gut microbiota has not been studied in PD patients.

In the present study, we conducted a comprehensive analysis of plasma BCAA and AAA profiles and the gut microbiota to define the interaction between the gut microbiota and metabolites and their roles in the development of PD.

## Results

### Demographics and clinical characteristics of study participants

Demographics and clinical characteristics of study participants are summarized in Table 1. A total of 106 PD patients (48 in early stage, 58 in advanced stage) and 114 controls were enrolled in this study. Age, sex, and body mass index (BMI) were indistinguishable between PD patients and controls. PD patients had an average Hoehn and Yahr stage (H&Y stage) of 2.5 ± 0.9, disease duration of 6.5 ± 4.6 years, and Movement Disorder Society-sponsored revision of the Unified Parkinson’s Disease Rating Scale (MDS-UPDRS) total score of 61.9 ± 21.8 points. All PD patients were using antiparkinsonian medications. Dopamine agonists were used by 70 patients, including pramipexole (*n* = 55), piribedil (*n* = 14), and ropinirole (*n* = 1). Monoamine oxidase-B (MAO-B) inhibitors, including selegiline (*n* = 34) and rasagiline (*n* = 2), were used by 36 patients. There were no significant differences in age, sex, or BMI between patients with early and advanced PD (see “METHODS”). The disease duration was longer; MDS-UPDRS total and part II, III, and IV scores were higher; and levodopa daily dose and levodopa equivalent daily dose (LEDD) was larger in patients with advanced stage. More patients were treated with levodopa in the advanced stage.

**Table 1:** Demographics and clinical characteristics of study participants.

	Controls (*n* = 114)	PD (*n* = 106)	Early PD (*n* = 48)	Advanced PD (*n* = 58)	*P* value^a^	*P* value^b^
Age (years)	68.0 ± 6.4	67.9 ± 6.5	66.8 ± 7.3	68.9 ± 5.7	0.972	0.101
Female, *n* (%)	48 (42.1)	47 (44.3)	24 (50.0)	23 (39.7)	0.786	0.329
BMI (kg/m^2^)	23.4 ± 1.6	22.9 ± 3.1	22.8 ± 3.2	23.0 ± 2.9	0.136	0.786
H&Y stage	n/a	2.5 ± 0.9	1.8 ± 0.4	3.2 ± 0.6	n/a	<0.001
Disease duration (years)	n/a	6.5 ± 4.6	3.9 ± 3.2	8.6 ± 4.5	n/a	<0.001
MDS-UPDRS total	n/a	61.9 ± 21.8	54.6 ± 17.8	68.0 ± 23.0	n/a	0.001
MDS-UPDRS I	n/a	12.9 ± 4.7	12.0 ± 4.4	13.6 ± 4.9	n/a	0.076
MDS-UPDRS II	n/a	14.1 ± 5.7	11.8 ± 4.1	15.9 ± 6.1	n/a	<0.001
MDS-UPDRS III	n/a	33.2 ± 13.6	30.1 ± 12.3	35.8 ± 14.2	n/a	0.030
MDS-UPDRS IV	n/a	1.8 ± 3.0	0.7 ± 2.1	2.6 ± 3.4	n/a	<0.001
Constipation, *n* (%)	n/a	92 (86.8)	39 (81.3)	53 (91.4)	n/a	0.155
Wexner score	n/a	14.2 ± 4.5	14.6 ± 4.9	13.8 ± 4.1	n/a	0.856
Medication for PD						
Levodopa, *n* (%)	n/a	97 (91.5)	40 (83.3)	57 (98.3)	n/a	0.010
Levodopa daily dose (mg)	n/a	433.7 ± 265.6	357.8 ± 272.6	496.6 ± 244.5	n/a	0.005
Dopamine agonist, *n* (%)	n/a	70 (66)	30 (62.5)	39 (67.2)	n/a	0.684
COMT inhibitor, *n* (%)	n/a	13 (12.3)	5 (10.4)	8 (13.8)	n/a	0.768
MAO-B inhibitor, *n* (%)	n/a	36 (34.0)	14 (29.2)	22 (37.9)	n/a	0.412
Trihexyphenidyl, *n* (%)	n/a	8 (7.5)	4 (8.3)	4 (6.9)	n/a	1.000
Amantadine, *n* (%)	n/a	13 (12.3)	7 (14.6)	6 (10.3)	n/a	0.562
LEDD (mg/day)	n/a	538.2 ± 292.6	447.0 ± 285.7	613.7 ± 278.5	n/a	<0.001

Data are shown as mean ± standard deviation or *n* (%). Comparisons between groups were assessed with the Student’s *t* test or Mann–Whitney U test for quantitative variables, and Fisher’s exact tests for categorical variables. Early PD was defined by an H&Y stage < 2.5, and advanced PD was defined by an H&Y stage ≥ 2.5.

*PD* Parkinson’s disease, *BMI* body mass index, *H&Y stage* Hoehn and Yahr stage, *MDS-UPDRS* Movement Disorder Society-sponsored revision of the Unified Parkinson’s Disease Rating Scale, *COMT* catechol-O-methyl transferase, *MAO-B* monoamine oxidase-B, LEDD, levodopa equivalent daily dose.

^a^Controls vs. PD.

^b^Early PD vs. Advanced PD.

### Profiles of plasma BCAAs and AAAs and their correlations with clinical characteristics and the gut microbiota in PD patients

After correction for covariates, the plasma Leu (1.2 ± 0.5 vs. 1.4 ± 0.7 μg/mL, *P* = 0.015), Ile (0.8 ± 0.4 vs. 1.0 ± 0.5 μg/mL, *P* = 0.031), Val (2.0 ± 0.6 vs. 2.5 ± 1.2 μg/mL, *P* < 0.001), and Tyr (11.0 ± 3.0 vs. 13.5 ± 6.7 μg/mL, *P* < 0.001) were significantly lower in PD patients compared with controls, while no differences were observed in Phe (1.1 ± 0.5 vs. 1.7 ± 4.0 μg/mL, *P* = 0.099) (Table 2).

**Table 2:** Plasma levels of BCAAs and AAAs in controls and PD patients.

	Controls (*n* = 114)	PD (*n* = 106)	Early PD (*n* = 48)	Advanced PD (*n* = 58)	*P* value^a^	*P* value^b^
*BCAAs*						
Leu (μg/mL)	1.4 ± 0.7	1.2 ± 0.5	1.4 ± 0.5	1.1 ± 0.4	0.015	0.001
Ile (μg/mL)	1.0 ± 0.5	0.8 ± 0.4	1.0 ± 0.4	0.7 ± 0.4	0.031	0.003
Val (μg/mL)	2.5 ± 1.2	2.0 ± 0.6	2.2 ± 0.7	1.7 ± 0.5	<0.001	0.001
*AAAs*						
Phe (μg/mL)	1.7 ± 4.0	1.1 ± 0.5	1.3 ± 0.5	0.9 ± 0.5	0.099	0.002
Tyr (μg/mL)	13.5 ± 6.7	11.0 ± 3.0	11.7 ± 3.4	10.5 ± 2.6	<0.001	0.010

Data are shown as mean ± standard deviation. Differences in BCAAs and AAAs between PD patients and controls were evaluated using ANCOVA, adjusting for age, sex, and BMI. Differences in BCAAs and AAAs between early and advanced PD patients were evaluated, adjusting for age, sex, BMI, levodopa (use or no use), and LEDD. Early PD was defined by an H&Y stage < 2.5, and advanced PD was defined by an H&Y stage ≥ 2.5.

*PD* Parkinson’s disease, *BCAAs* branched-chain amino acids, *Leu* leucine, *Ile* isoleucine, *Val* valine, *AAAs* aromatic amino acids, *Phe* phenylalanine, *Tyr* tyrosine, *ANCOVA* analysis of covariance, *BMI* body mass index, *LEDD* levodopa equivalent daily dose.

^a^Controls vs. PD.

^b^Early PD vs. Advanced PD.

**Fig. 1:**
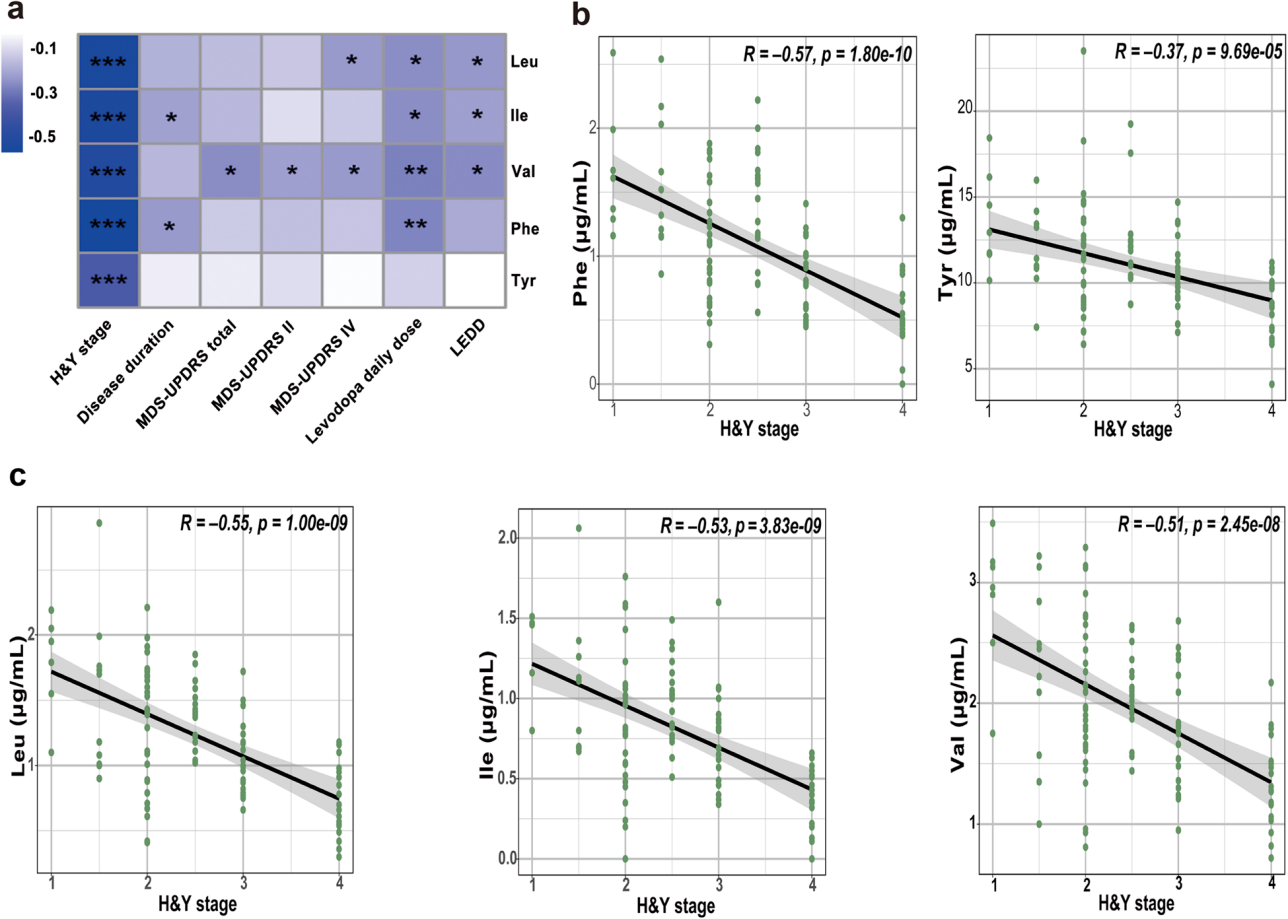
Correlations between plasma BCAAs and AAAs and PD clinical characteristics. The heat maps represent Spearman’s rank correlations of BCAAs and AAAs with PD clinical characteristics (**a**). Correlation coefficients are represented by gradient colors. *P < 0.05, **P < 0.01, and ***P < 0.001. Scatter plot of plasma AAAs (**b**) and BCAAs (**c**) vs. H&Y stage. BCAAs, branched-chain amino acids; AAAs, aromatic amino acids; Leu, leucine; Ile, isoleucine; Val, valine; Phe, phenylalanine; Tyr, tyrosine; MDS-UPDRS, Movement Disorder Society-sponsored revision of the Unified Parkinson’s Disease Rating Scale; LEDD, levodopa equivalent daily dose; H&Y stage, Hoehn and Yahr stage.

We performed Spearman’s rank correlation analysis to explore the relationship of plasma BCAAs and AAAs with clinical characteristics (Fig. 1a). All of the AAAs and BCAAs negatively correlated with H&Y stage (Fig. 1b, c), including Phe (*R* = −0.57, *P* = 1.80E−10), Tyr (*R* = −0.37, *P* = 9.69E−05), Leu (*R* = −0.55, *P* = 1.00E−09), Ile (*R* = −0.53, *P* = 3.83E−09), and Val (*R* = −0.51, *P* = 2.45E−08). Leu was negatively associated with MDS-UPDRS IV score (*R* = −0.22, *P* = 0.025), levodopa daily dose (*R* = −0.24, *P* = 0.013), and LEDD (*R* = −0.22, *P* = 0.021). Ile was negatively correlated with disease duration (*R* = −0.20, *P* = 0.040), levodopa daily dose (*R* = −0.24, *P* = 0.012), and LEDD (*R* = −0.21, *P* = 0.033). Val was negatively associated with MDS-UPDRS total score (*R* = −0.25, *P* = 0.011), MDS-UPDRS II score (*R* = −0.21, *P* = 0.034), MDS-UPDRS IV score (*R* = −0.22, *P* = 0.025), levodopa daily dose (*R* = −0.27, *P* = 0.005), and LEDD (*R* = −0.25, *P* = 0.011). Phe was negatively correlated with disease duration (*R* = −0.22, *P* = 0.026) and levodopa daily dose (*R* = −0.26, *P* = 0.007). However, we found no significant difference in plasma BCAAs or AAAs in patients treated with or without other antiparkinsonian medications (Supplementary Table 1).

To see if plasma BCAAs and AAAs were independently correlated with PD severity (based on H&Y stage), the analysis of confounding factors was performed using the analysis of covariance (ANCOVA) method. After correcting for covariates, the independent associations of plasma BCAAs and AAAs with PD severity remained significant. In addition, plasma Phe and Tyr were correlated with levodopa daily dose during the ANCOVA analysis (Supplementary Table 2).

We further identified whether the disturbances of plasma BCAAs and AAAs were associated with the gut microbiota in PD patients. There were 46 microbial taxa (family and genus levels) associated with BCAAs and/or AAAs (e.g., *Erysipelotrichaceae*, *Desulfovibrionaceae*, *Acidaminococcaceae*, and *Streptococcaceae* at the family level and *Lactobacillus* and *Streptococcus* at the genus level) (*P* < 0.05, Supplementary Fig. 1a, b, Supplementary Table 3). After correction of multiple-hypothesis testing, family *Desulfovibrionaceae*, *Acidaminococcaceae*, and *Erysipelotrichaceae* were significantly correlated with BCAAs and/or AAAs with the Benjamini–Hochberg false-discovery rate (FDR)-*P* < 0.05 (Supplementary Fig. 1a, Supplementary Table 3).

### Differences in plasma BCAAs and AAAs and microbiota composition between early and advanced PD patients

As plasma BCAAs and AAAs were correlated with H&Y stage and the gut microbiota, and the gut microbiota was also associated with H&Y stage^21^, we hypothesized that microbiota composition may become altered during the shift from early to advanced PD, leading to the exacerbated dysregulation of plasma amino acids. We thus further explored the potential influence of the gut microbiota on plasma BCAAs and AAAs in early and advanced PD according to H&Y stage.

**Fig. 2:**
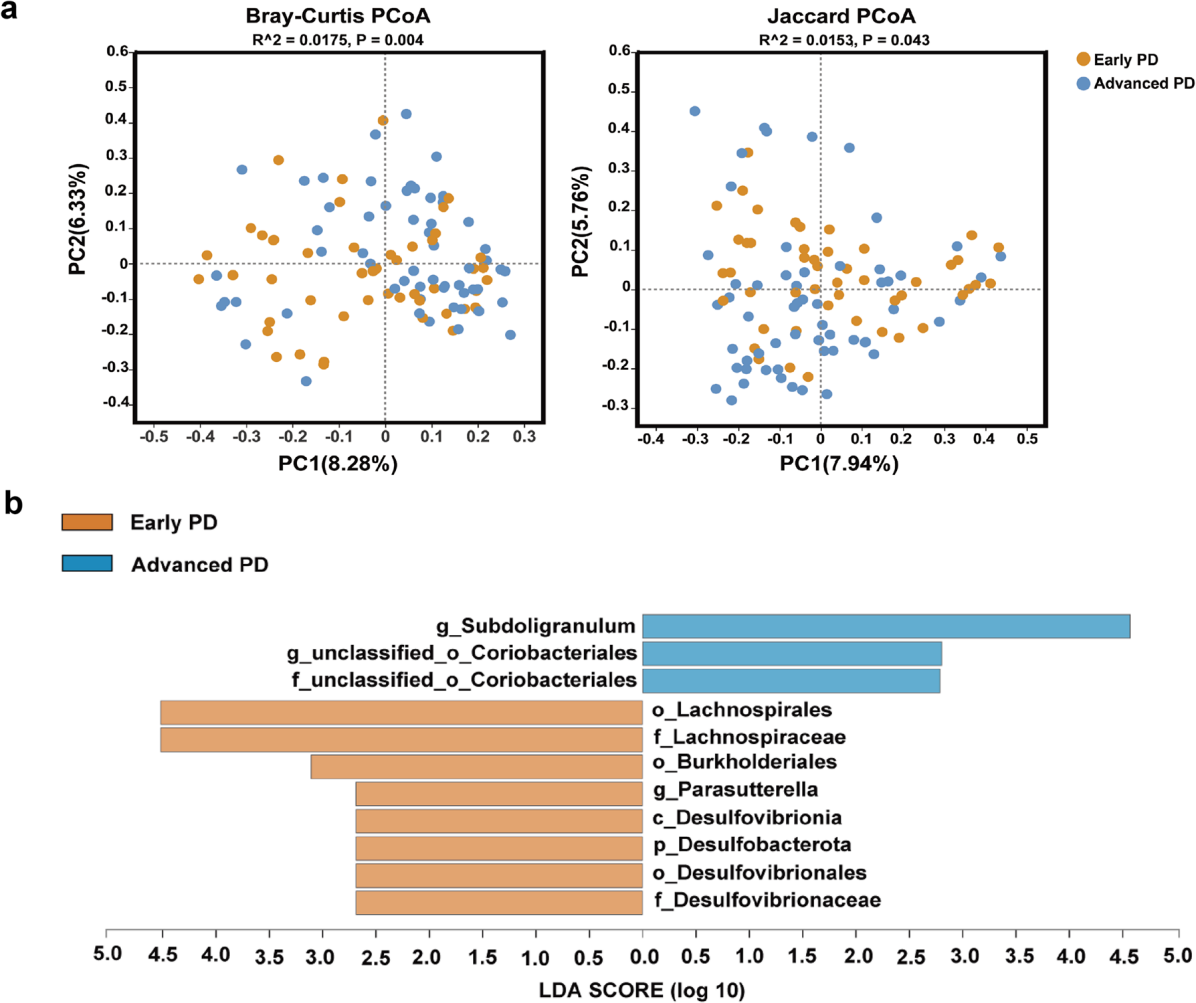
The alteration of fecal microbiota between early and advanced PD patients. **a** Beta diversity plots to visualize the difference in microbiota structure between early and advanced PD patients. PCoA plots show the beta-diversity with Bray–Curtis and Jaccard measures. **b** LEfSe analysis revealed remarkable microbial differences between early and advanced PD patients, adjusting for age, sex, BMI, levodopa (use or no use), levodopa daily dose, and LEDD. *Abbreviations:* PCoA, principal coordinates analysis; LEfSe, linear discriminant analysis (LDA) effect size; p, phylum; c, class; o, order; f, family; g, genus; BMI, body mass index; LEDD, levodopa equivalent daily dose.

As expected, plasma BCAAs and AAAs were significantly lower in patients with advanced PD (Table 2). To explore whether decreased plasma BCAAs and AAAs were associated with alterations of microbiota composition, we compared the gut microbial richness and composition, including alpha-diversity and beta-diversity between early and advanced PD patients. Among the commonly used alpha-diversity indices, abundance-based coverage estimator (ACE) (*P* = 0.522), Chao1 (*P* = 0.522), and Shannon (*P* = 0.083) were not significantly different, except for Simpson (*P* = 0.048). Differences were found in beta-diversity based on the Bray–Curtis (*R*^2^ = 0.0175, *P* = 0.004) and Jaccard (*R*^2^ = 0.0153, *P* = 0.043) metrics (Fig. 2a). Furthermore, a total of 18 microbial taxa at all levels with different abundances were identified in early and advanced PD patients according to LEfSe analysis (Supplementary Fig. 2). Since levodopa can greatly influence bacterial composition^22–24^, we further evaluated the alteration in the abundance of microbial taxa between early and advanced PD patients adjusting for levodopa usage and other confounding factors, and a total of 11 microbial taxa were finally identified (Fig. 2b, Supplementary Table 4).

We then investigated the relationships of BCAAs and AAAs with the 11 microbial taxa in patients with early and advanced PD, respectively. A total of 5 microbial taxa were associated with BCAAs and/or AAAs (*P* < 0.05, Supplementary Table 5). After correction of multiple-hypothesis testing, phylum *Desulfobacterota*, class *Desulfovibrionia*, order *Desulfovibrionales*, and family *Desulfovibrionaceae*, which were more abundant in the early stage of PD compared with the advanced stage, showed significantly positive association with Phe (FDR-*P* < 0.05, Supplementary Table 5), and these associations remained significant adjusting for confounding factors (Supplementary Table 6).

### Differences in microbiota functional profiling between early and advanced PD patients

**Fig. 3:**
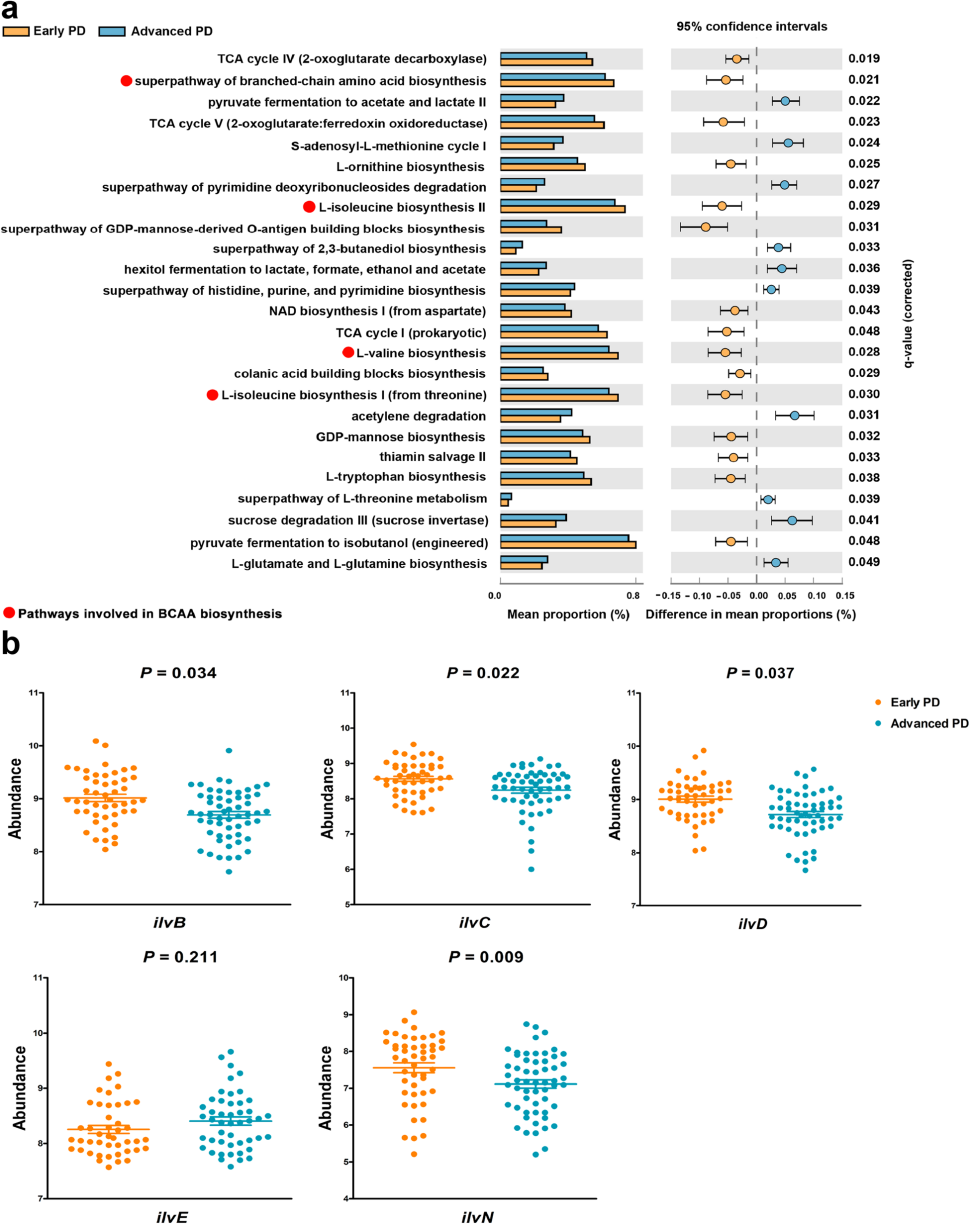
Differences in microbiota functional profiling between early and advanced PD patients. **a** Predicted functional analysis identified 25 pathways with significantly different abundances of predicted genes between early and advanced PD patients. Pathways associated with BCAA biosynthesis had fewer numbers of predicted genes in patients with advanced PD, relative to early PD patients. Predicted functional microbiota profiling was performed using PICRUSt2. The abundances of predicted genes in metabolic pathways were compared using White’s nonparametric t-test with FDR correction using the STAMP software. **b** Comparisons of critical gene abundances in fecal samples between early and advanced PD patients. The gene abundances were expressed as log_10_ copy number per gram of dry weight feces. Differences between groups were assessed using ANCOVA, adjusting for age, sex, BMI, levodopa (use or no use), and LEDD. Data are presented as mean ± SEM. *Abbreviations:* PICRUSt2, Phylogenetic Investigation of Communities by Reconstruction of Unobserved States 2; FDR, Benjamini–Hochberg false-discovery rate; STAMP, Statistical Analysis of Metagenomic Profiles; ANCOVA, analysis of covariance; LEDD, levodopa equivalent daily dose; SEM, standard error of the mean.

To further investigate the potential mechanistic links between the gut microbiota and plasma amino acids, predictive functional analysis was performed. We employed Phylogenetic Investigation of Communities by Reconstruction of Unobserved States 2 (PICRUSt2), a computational tool that allows the use of 16S rRNA amplicon data to predict genes, to calculate their abundances, assign them to metabolic pathways using the MetaCyc database, and test the differences between early and advanced PD patients. We identified 106 microbial MetaCyc metabolic pathways that had different abundances of predicted genes between early and advanced PD patients, among which 23 pathways were associated with amino acid metabolism (*P* < 0.05, Supplementary Table 7). After correction of multiple-hypothesis testing, there were 25 metabolic pathways that had significantly different abundances of predicted genes between early and advanced PD patients, among which 9 pathways were associated with amino acid metabolism (FDR-*P* < 0.05, Fig. 3a, Supplementary Table 7). Notably, four pathways involved in BCAA biosynthesis had fewer numbers of predicted genes in patients with advanced PD compared with patients in the early stage. To verify the alteration of BCAA biosynthesis pathways identified in the predictive functional analysis, we quantified the fecal abundances of critical genes (*ilvB*, *ilvC*, *ilvD*, *ilvE*, and *ilvN*)^25–28^ responsible for BCAA biosynthesis. The abundances of *ilvB*, *ilvC*, *ilvD*, and *ilvN* were decreased in patients with advanced PD (Fig. 3b). Additionally, we measured BCAA concentrations in fecal samples from 86 PD patients (42 early stage vs. 44 advanced stage). As expected, the fecal concentrations of BCAAs were significantly lower in advanced PD patients (Supplementary Fig. 3).

## Discussion

In this study, we found that plasma BCAAs (Leu, Ile, and Val) and Tyr among the AAAs were decreased in PD patients. More importantly, BCAAs and AAAs were negatively associated with H&Y stage. Compared with early PD patients, BCAA and AAA levels were declined further and the microbial composition was altered in patients with advanced PD. Eleven microbial taxa with different abundances between early and advanced stages of PD were identified. Predictive functional analysis indicated that the numbers of predicted genes in four pathways involved in BCAA biosynthesis were significantly lower in the advanced stage of PD. What’s more, the fecal abundances of critical genes (*ilvB*, *ilvC*, *ilvD*, and *ilvN*) involved in BCAA biosynthesis were decreased and the fecal concentrations of BCAAs were lower in advanced PD patients.

Several studies have explored the changes of BCAAs and AAAs in peripheral blood of PD patients. Serum BCAAs from 17 PD patients in northern India were found to be higher using (1)H nuclear magnetic resonance (NMR) spectroscopy^10^. A study from Sweden with 22 PD patients showed unchanged plasma BCAAs levels^11^ as detected by liquid chromatography-mass spectrometry (LC-MS). Plasma Tyr levels measured by ionic exchange chromatography were found to be higher in 31 PD patients from Spain^12^, or similar with those in controls reported in Japan^5,13^. In this study, we measured plasma BCAA and AAA levels using high-performance liquid chromatography with fluorescence detection (HPLC-FLD) in a large cohort of 106 PD patients and 114 controls and found that plasma BCAAs and Tyr were decreased in PD patients. Similarly, Molina et al. reported that plasma Val and Leu levels were reduced in PD patients^12^, consistent with our findings. In addition, lower levels of Val and Tyr have also been reported in other neurological degenerative disorders^29,30^.

The metabolic disturbances of plasma amino acids in PD patients could be related to several factors. Increased energy expenditure^31^ in PD patients may accelerate the consumption of plasma amino acids. BCAAs and AAAs in the human body are mainly derived from dietary nutrients, so gastrointestinal dysfunction in PD^32^ could disturb the absorption of these amino acids. Notably, the gut microbiota is a key factor in shaping the metabolic profiles of dietary ingredients^33^. PD patients suffer from gut microbiota dysbiosis^17–20^, which may disturb the metabolism of BCAAs and AAAs and is linked with alterations of these amino acids in plasma. Indeed, we found correlations between plasma BCAAs and AAAs and several microbial taxa (e.g., negative correlations with *Streptococcaceae*, *Streptococcus*, and *Lactobacillus*). Notably, several species and strains of *streptococci* consume amino acids (Leu, Ile, Val, and Tyr) for growth^34^. The genus *Lactobacillus* could produce enzymes responsible for the catabolism of several amino acids (e.g., BCAAs, AAAs, aspartic acid, and glutamic acid)^35^. After correction of multiple-hypothesis testing, *Desulfovibrionaceae*, *Acidaminococcaceae*, and *Erysipelotrichaceae* showed significant correlations with BCAAs or AAAs. Specific genera in *Desulfovibrionaceae* (e.g., *Desulfovibrio desulfuricans*) harbor genes responsible for amino acid metabolism^36^. *Acidaminococcaceae* has been reported to ferment glutamate to produce 2-hydroxyglutarate, which could be further metabolized to 2-ketoglutarate^37^, which is involved in the biosynthesis of BCAAs^38^. *Erysipelotrichaceae*, which was negatively correlated with Leu and Phe, has been reported to be more abundant in PD patients compared with healthy controls^39^. *Erysipelotrichaceae* plays an important role in nutrition metabolism^40^, which may affect the content of amino acids in the human body. These findings indicate that the gut microbiota may be related to the alteration of plasma BCAAs and AAAs in PD patients.

Meanwhile, we found that lower plasma BCAA and AAA levels were correlated with longer disease duration, higher MDS-UPDRS total, part II, and IV scores, larger levodopa daily dose and LEDD, and especially correlated with more severe disease conditions (H&Y stage). Levodopa, the commonly used antiparkinsonian drug, could compete with BCAAs and AAAs in utilizing the stereospecific transport system in the small intestine^41^; thus, a larger levodopa dose may compromise the absorption of BCAAs and AAAs, resulting in lower plasma levels. However, we found only Phe and Tyr levels were associated with levodopa daily dose adjusting for confounding factors. Notably, we found that PD severity was significantly correlated with plasma BCAAs and AAAs after correction for covariates. The negative correlations of BCAAs and AAAs with PD severity could be partly accounted for by worse gastrointestinal dysfunction^42^ and higher energy expenditure^43^ in the advanced stage. More importantly, studies have observed dynamic shifts in microbial composition and metabolites during disease progression, and disease progression may be influenced by the metabolic output of the gut microbiota^44,45^. The gut microbiota has been reported to be correlated with PD severity and disease duration^21^; thus, we supposed that alterations in the microbiota community from the early to advanced stage of disease may be related to the reductions of plasma BCAAs and AAAs. Indeed, compared with patients in the early stage of PD, the gut microbiota compositions changed and the abundances of 11 microbial taxa were altered in the advanced stage. Among the 11 microbial taxa, four microbial taxa belonging to phylum *Desulfobacterota* were more abundant in early stage and showed significantly positive correlations with Phe. *Desulfobacterota*, a phylum known for sulfate reduction, is capable of expressing genes responsible for nitrogen fixation, and the fixed nitrogen could be used for amino acid biosynthesis^46^. Functional predictive analysis revealed that nine pathways involved in amino acid metabolism had different abundances of predicted genes between early and advanced PD patients. Notably, pathways involved in BCAA biosynthesis had fewer numbers of predicted genes in the advanced stage. The BCAA biosynthesis pathways in microorganisms have four key enzymes: acetohydroxy acid synthase (AHAS, encoded by *ilvB* and *ilvN*), acetohydroxy acid isomeroreductase (AHAIR, encoded by *ilvC*), dihydroxyacid dehydratase (DHAD, encoded by *ilvD*), and transaminase (TA, encoded by *ilvE*)^25–28^. Thus, *ilvB*, *ilvC*, *ilvD*, *ilvE*, and *ilvN* are critical genes in BCAA biosynthesis pathways, and downregulation of these genes results in the reduction of BCAA biosynthesis^27,28,47,48^. The decreased expression levels of *ilvB*, *ilvC*, *ilvD*, and *ilvN* genes further validated the reduction of BCAA biosynthesis in advanced PD patients. Furthermore, we used a more straightforward approach to validate the alteration of BCAA biosynthesis pathways by measuring fecal BCAA concentrations, and we found that fecal BCAA concentrations were decreased in advanced PD patients. We suppose from our findings that the reduced biosynthesis of amino acids by bacteria in the gut could decrease the content of amino acids for absorption, which may be associated with their reduction in plasma.

BCAAs, the essential amino acids necessary for protein synthesis and nitrogen donors for the synthesis of neurotransmitters, play an important role in normal functioning of central nervous system^7^. BCAAs participate in the metabolism of glutamate, the most abundant excitatory neurotransmitter in the brain. Abnormal glutamate metabolism is commonly observed in neurodegenerative disorders, which is partially attributed to the deficiency of the enzyme glutamate dehydrogenase^49,50^. BCAAs can activate glutamate dehydrogenase^8^, which contributes to the modification of glutamate metabolism and glutamatergic transmission, to reduce the toxic effect of glutamate. The depletion of dopamine in the brain is closely involved in PD^51^. Phe and Tyr serve as the critical substrates for the production of dopamine. Phe is enzymatically hydroxylated by phenylalanine hydroxylase to yield Tyr, which is further hydroxylated by tyrosine hydroxylase to produce dopa, the precursor of dopamine^5^. BCAAs supplementation can have beneficial effects in patients with neurological disorders. Amyotrophic lateral sclerosis (ALS) patients exhibit slower disease progression after oral administration of a BCAA mixture^52^. According to our results, the deficiency of BCAAs and AAAs occurred in PD patients and correlated with disease severity, so we propose that supplementation with these amino acids or restoration of them through microbiota manipulation may have beneficial effects and serve as a promising approach for the treatment of PD.

The present study has some limitations. (1) Although participants with high- or low-amino acid diets were excluded and fasting plasma samples were used for analysis, the information regarding dietary characteristics was not well evaluated. (2) A follow-up study is needed to better understand the relationship between plasma amino acid concentrations and disease progression. (3) Metagenome shotgun sequencing, which can provide more detailed information about the gut microbiota, is also needed to learn more about the microbial species-amino acid interaction. (4) From our results, we cannot completely exclude the influence of levodopa and other medications on gut microbiota and metabolites. In addition, it is difficult to say which is the initial power to drive the changes of amino acids and gut microbiota due to the complex interaction between them. In the future, it is better to explore the interactions between amino acids, gut microbiota and clinical characteristics in de novo PD patients.

Overall, we found that the disturbances of plasma BCAAs and AAAs in PD patients may be related to the gut microbiota and exacerbated with PD severity. These findings are beneficial for a better understanding of the relationship between the gut microbiota and host metabolism, and it may be necessary to introduce therapy of PD by focusing on metabolites related to the gut microbiota in the future.

## Methods

### Participant recruitment and data collection

One hundred six individuals with PD were recruited from the Movement Disorders Clinic at the Department of Neurology and Institute of Neurology, Ruijin Hospital. PD was diagnosed according to the United Kingdom Parkinson’s Disease Society Brain Bank criteria^53^. Exclusion criteria for PD patients were: (1) atypical or secondary parkinsonism, (2) serious chronic illnesses (e.g., diabetes, heart failure, liver cirrhosis, malignancy, hematological or autoimmune diseases, or inflammatory gastrointestinal disease), (3) the use of probiotic or antibiotic supplements for the three months before enrollment, or (4) a high- or low-amino acid diet.

One hundred fourteen controls matched by age, sex, and nutritional status (BMI) were recruited over the same period. Inclusion criteria for controls were: (1) normal physical exam, (2) no digestive symptoms or disease, (3) no neurodegenerative disease, and (4) avoidance of a high- or low-amino acid diet. Exclusion criteria for controls were the same as for PD patients. This study was approved by the Research Ethics Committee, Ruijin Hospital, Shanghai Jiao Tong University School of Medicine, Shanghai, China. All participants were informed of the purpose of this study and provided written informed consent.

Demographics of age, sex, height, weight, and BMI were obtained for all participants. Clinical data were obtained through face-to-face interviews with movement disorder specialists. Motor function was evaluated using the H&Y stage^54^ and MDS-UPDRS^55^. Disease duration from onset to study beginning and medication history were recorded. LEDD was calculated using a method reported in a previous study^56^. PD severity was categorized by H&Y stage^57^; early PD was defined by an H&Y stage <2.5, and advanced PD was defined by an H&Y stage ≥2.5.

Venous blood samples were taken after an overnight fast of at least 10 h from PD patients and controls, and immediately centrifuged at 4 °C to obtain plasma, which was further stored at −80 °C until analysis. Fecal samples were collected from PD patients in sterile ﻿fecal collection containers and stored at −80 °C prior to processing.

### Analysis of plasma BCAAs and AAAs

Concentrations of plasma BCAAs and AAAs were measured by HPLC-FLD. Briefly, the plasma amino acids were derivatized, then separated with a YMC-C18-EXRS column (150 mm × 4.6 mm, 3 µm). The amino acids were fluorescently detected with excitation and emission wavelengths of 260 and 325 nm, respectively. The established method was validated by determining linearity, precision, accuracy, limits of detection (LODs), and limits of quantification (LOQs), as previously described^58,59^. The linearity was evaluated by preparing standard mixtures at seven different concentrations, which were analyzed based on the methods described above. Then, the calibration curves were established by plotting the peak area of BCAAs and AAAs versus their respective concentrations in the calibration samples. The concentration range and the coefficient of determination (R^2^) for each analyte were determined. The percentage of relative standard deviation (RSD) was determined as a measurement of interday and intraday precision. Deviation from the true value was determined by comparing the obtained concentration with the nominal concentration for interday and intraday accuracy and expressed as % accuracy^59^. The LODs were determined using the lowest concentration with a peak area of signal-to-noise (S/N) ratio of ≥3. The LOQs were referred to as the lowest concentration on a calibration curve at which quantitative results can be reported with a high degree of confidence that produced a peak with an S/N ratio of ≥10 (Supplementary Table 8).

### 16S rRNA gene amplification and sequencing

DNA extraction from thawed fecal samples was performed using the QIAamp DNA Stool Mini Kit (Qiagen, Hilden, Germany) following the manufacturer’s instructions. The V3–V4 regions of 16S rRNA genes were amplified by polymerase chain reaction (PCR) using the barcoded primers 341F 5′-CCTACGGGRSGCAGCAG-3′ and 806R 5′-GGACTACVVGGGTATCTAATC-3′. PCR reactions were performed in 30 μL mixtures containing 15 μL of 2 × KAPA Library Amplification ReadyMix, 1 μL of each primer (10 μM), and 50 ng of template DNA and ddH_2_O. The procedure of PCR was as follows: 95 °C for 3 min, followed by 30 cycles at 98 °C for 20 s, 58 °C for 15 s, and 72 °C for 20 s, then a final extension at 72 °C for 5 min. Amplicons were extracted from 2% agarose gels and purified using the AxyPrep DNA Gel Extraction Kit (Axygen Biosciences, Union City, CA, USA) according to the manufacturer’s instructions and were quantified using Qubit^®^2.0 (Invitrogen, Carlsbad, CA, USA). The pooled library was sequenced using an Illumina MiSeq system (Illumina, Inc., San Diego, CA, USA).

### Microbiota data analysis

Bacterial 16 S rRNA gene sequence data were demultiplexed and quality-filtered, and chimeric sequences were removed using the Divisive Amplicon Denoising Algorithm 2 (DADA2)^60^ with the open-source software Quantitative Insights Into Microbial Ecology 2 (QIIME2)^61^ (version 2020.2). Taxonomical assignment was performed using the SILVA database (version 138)^62^. Alpha diversity indices, including Chao1, Shannon, Simpson, and ACE, were generated using mothur (version 1.35.1)^63^. For beta-diversity, the dissimilarities (distances) were calculated using the vegan package in the R software (version 4.0.3; R Foundation for Statistical Computing, Vienna, Austria). To avoid the bias of the metric selection, we calculated the distances using the Jaccard and Bray–Curtis dissimilarity metrics. These metrics were also used for ordination by principal coordinates analysis (PCoA), which was performed for all dimension reduction analyses. Significant differences were assessed using permutational multivariate analysis of variance (PERMANOVA)^64^ with 999 permutations. Linear discriminant analysis (LDA) effect size (LEfSe) analysis with an alpha cutoff of 0.05 and an effect size cutoff of 2.5 was performed to estimate the effect size of each differentially abundant feature. Predicted functional microbiota profiling was performed using PICRUSt2 (version 2.2.0-b)^65^. The metabolic pathways were annotated by the MetaCyc database (version 23.1)^66^, and differences between groups were identified using White’s nonparametric t-test with FDR correction^67^ using the Statistical Analysis of Metagenomic Profiles (STAMP) software (version 2.1.3)^68^.

### Quantitative real-time PCR

Quantitative real-time PCR was performed as previous described with some modifications^69^. TIANamp Stool DNA Kit (TIANGEN Biotech Co. Ltd., Beijing, China) was used to extract fecal bacterial DNA, in accordance with the manufacturer’s instructions. Quantitative real-time PCR with SYBR Green I was performed in a LineGene FQD-96A Sequence Detection System (Hangzhou Bioer Technology Co. Ltd., Hangzhou, China). The Primer Premier 5.0 software was used to develop specific primers for *ilvB*, *ilvC*, *ilvD*, *ilvE*, and *ilvN* (Supplementary Table 9). All real-time PCR amplifications were carried out in a total volume of 20 μL per reaction mixture, containing 1 μL of template genomic DNA (gDNA), 10 μL 2 × T5 Fast qPCR Mix (SYBR Green I), and 10 μM of the primer. The amplification consisted of an initial incubation at 95 °C for 1 min, followed by 40 cycles at 95 °C for 15 s, 60 °C for 15 s, and 72 °C for 30 s. Plasmid DNA standards were constructed by TA cloning using the pClone007 Versatile Simple Vector Kit (TsingKe Biological Technology Co., Beijing, China) for absolute quantification. The plasmid DNA was extracted with the Plasmid DNA Extraction Mini Kit (TsingKe Biological Technology Co., Beijing, China) and quantified with the Epoch microplate spectrophotometer (BioTek Instruments, Winooski, VT, USA). Serial dilutions of the standard were used as templates to construct the real-time PCR standard curve. All quantifications were performed in triplicate, and the mean Ct was used to calculate the copies of *ilvB*, *ilvC*, *ilvD*, *ilvE*, and *ilvN*. The abundances of *ilvB*, *ilvC*, *ilvD*, *ilvE*, and *ilvN* were expressed as log_10_ copy number per gram of dry weight feces.

### Liquid chromatography-tandem mass spectrometry (LC-MS) analysis for fecal BCAAs

LC-MS analyses were performed using an ultra-performance liquid chromatography coupled to tandem mass spectrometry (UPLC-MS) system (ACQUITY UPLC-Xevo TQ-S; Waters Corp., Milford, MA, USA) as previously described^70^. Briefly, the freeze-dried fecal samples from 86 PD patients (42 early stage vs. 44 advanced stage) were thawed in an ice-bath, and about 5 mg of each sample was weighted. Then, 20 μL of ultrapure water was added, and 120 μL of methanol containing internal standards solution was added to extract the metabolites. The samples were homogenated for 3 min and centrifuged at 13500 g for 10 min. Then, 30 μL of supernatant was transferred to a 96-well plate for derivatization using the Biomek 4000 workstation (Biomek 4000; Beckman Coulter, Inc., Brea, CA, USA). After derivatization, 400 μL of ice-cold 50% methanol solution was added to dilute the samples. Then, the plate was stored at –20 °C for 20 min and centrifugated at 4000 *g* for 30 min. Next, 135 μL of supernatant was transferred to a new 96-well plate for LC-MS analysis. The raw data files generated by LC-MS were processed using the MassLynx software (version 4.1; Waters, Milford, MA, USA) to perform peak integration, calibration, and quantitation for each metabolite.

### Statistical analysis

The statistical analysis was performed using the SPSS software (version 22.0, IBM Corporation, Armonk, NY, USA) and R software (version 4.0.3; R Foundation for Statistical Computing, Vienna, Austria). Comparisons between clinical variables were assessed with the Student’s *t* test or Mann–Whitney U test for quantitative variables, and Fisher’s exact tests for categorical variables. Differences in plasma BCAAs and AAAs between PD patients and controls were evaluated using ANCOVA, adjusting for age, sex, and BMI. Differences in plasma BCAAs and AAAs, bacterial taxa, fecal abundances of critical genes involved in BCAA biosynthesis, and fecal BCAA concentrations between early and advanced PD patients were evaluated adjusting for confounding factors. Spearman’s rank correlation was performed to evaluate the correlations of plasma amino acids with clinical characteristics and gut microbial taxa. In all cases of multiple-hypothesis testing, FDR-*P* < 0.05 was considered to be statistically significant. To perform exploratory analyses on the correlation between plasma BCAAs and AAAs and microbial taxa, the results of multiple-hypothesis testing with *P* < 0.05 are also presented and discussed.

### Reporting summary

Further information on research design is available in the Nature Research Reporting Summary linked to this article.

## Supplementary information

Supplementary information

Reporting Summary

## Data Availability

Sequences generated and analyzed during this study are accessible from the National Center for Biotechnology Information (NCBI) Sequence Read Archive (SRA) under the accession code SRP337726. The key data are included in this published article and its supplementary information files. Other datasets are available from the corresponding author upon reasonable request.
