# Supplementary material for: Plasma branched-chain and aromatic amino acids correlate with the gut microbiota and severity of Parkinson’s disease
Source: NPJ Parkinsons Dis. 2022 Apr 21;8:48. doi: 10.1038/s41531-022-00312-z (PMC9023571; doi:10.1038/s41531-022-00312-z)
Supplement: Supplementary file 1 — Supplementary information [file 41531_2022_312_MOESM1_ESM.pdf]

Supplementary Table 1. Comparisons of plasma BCAA and AAA levels between anti-parkinsonian medication users and non-users in PD patients

Medications		Leu (μg/mL)	Ile (μg/mL)	Val (μg/mL)	Phe (μg/mL)	Tyr (μg/mL)
Dopamine agonist	Users	1.2 ± 0.5	0.8 ± 0.4	1.9 ± 0.6	1.0 ± 0.5	11.1 ± 3.3
	Non-users	1.4 ± 0.5	0.9 ± 0.4	2.1 ± 0.6	1.2 ± 0.5	10.9 ± 2.4
	P value	0.097	0.173	0.203	0.107	0.754
COMT inhibitor	Users	1.2 ± 0.6	0.9 ± 0.5	1.8 ± 0.7	1.2 ± 0.6	11.3 ± 3.6
	Non-users	1.2 ± 0.5	0.8 ± 0.4	2.0 ± 0.6	1.1 ± 0.5	11.0 ± 3.0
	P value	0.996	0.618	0.495	0.363	0.768
MAO-B inhibitor	Users	1.3 ± 0.6	0.9 ± 0.4	2.1 ± 0.6	1.2 ± 0.6	10.9 ± 2.7
	Non-users	1.2 ± 0.4	0.8 ± 0.4	1.9 ± 0.6	1.0 ± 0.5	11.1 ± 3.2
	P value	0.153	0.302	0.121	0.087	0.817
Trihexyphenidyl	Users	1.3 ± 0.5	0.8 ± 0.3	2.0 ± 0.7	1.3 ± 0.6	11.3 ± 2.5
	Non-users	1.2 ± 0.5	0.8 ± 0.4	1.9 ± 0.6	1.1 ± 0.5	11.0 ± 3.1
	P value	0.488	0.990	0.746	0.195	0.757
Amantadine	Users	1.1 ± 0.5	0.7 ± 0.4	1.8 ± 0.7	0.9 ± 0.6	12.4 ± 4.0
	Non-users	1.2 ± 0.5	0.8 ± 0.4	2.0 ± 0.6	1.1 ± 0.5	10.8 ± 2.8
	P value	0.383	0.219	0.233	0.299	0.086

Data are presented as mean ± standard deviation. Differences between groups were assessed using Student's *t*-test for normally distributed variables, otherwise the Mann–Whitney U test.

Abbreviations: BCAAs, branched-chain amino acids; AAAs, aromatic amino acids; Leu, leucine; Ile, isoleucine; Val, valine; Phe, phenylalanine; Tyr, tyrosine; COMT, catechol-O-methyl transferase; MAO-B, monoamine oxidase-B.

Supplementary Table 2. ANCOVA analysis for plasma BCAAs and AAAs after correction of covariates

Amino acids	Factor	dF	F	*P* value
Leu	Group (Early PD VS Advanced PD)	1	11.961	0.001
	Sex (Male VS Female)	1	0.617	0.434
	Age	1	0.082	0.775
	BMI	1	0.118	0.732
	Levodopa (use or no use)	1	0.209	0.649
	Levodopa daily dose	1	0.365	0.547
	LEDD	1	0.023	0.880
Ile	Group (Early PD VS Advanced PD)	1	8.968	0.003
	Sex (Male VS Female)	1	0.042	0.839
	Age	1	0.249	0.619
	BMI	1	0.067	0.796
	Levodopa (use or no use)	1	0.508	0.478
	Levodopa daily dose	1	1.018	0.315
	LEDD	1	0.102	0.751
Val	Group (Early PD VS Advanced PD)	1	11.959	0.001
	Sex (Male VS Female)	1	0.021	0.885
	Age	1	0.076	0.783
	BMI	1	2.145	0.146
	Levodopa (use or no use)	1	0.030	0.864
	Levodopa daily dose	1	0.705	0.403
	LEDD	1	0.097	0.756
Phe	Group (Early PD VS Advanced PD)	1	9.944	0.002
	Sex (Male VS Female)	1	1.375	0.244
	Age	1	0.283	0.596
	BMI	1	1.08E-04	0.992
	Levodopa (use or no use)	1	0.238	0.627
	Levodopa daily dose	1	4.038	0.047
	LEDD	1	1.921	0.169
Tyr	Group (Early PD VS Advanced PD)	1	6.300	0.014
	Sex (Male VS Female)	1	3.645	0.059
	Age	1	2.642	0.107
	BMI	1	4.221	0.043
	Levodopa (use or no use)	1	1.690	0.197
	Levodopa daily dose	1	4.001	0.048
	LEDD	1	3.418	0.068

Abbreviations: ANCOVA, analysis of covariance; BCAAs, branched-chain amino acids; AAAs, aromatic amino acids; Leu, leucine; Ile, isoleucine; Val, valine; Phe, phenylalanine; Tyr, tyrosine; BMI, body mass index; LEDD, levodopa equivalent daily dose.

Supplementary Table 3. Correlations of plasma BCAAs and AAAs with the gut microbiota in PD patients

Amino acids	Bacterial Taxa	R	*P* value	FDR-*P* value
Leu	f_Erysipelotrichaceae	-0.3628	1.33E-04	2.48E-02
	f_Desulfovibrionaceae	0.3239	7.06E-04	5.83E-02
	f_Acidaminococcaceae	0.2979	1.93E-03	1.19E-01
	f_Muribaculaceae	0.2947	2.16E-03	1.19E-01
	f_Anaerofustaceae	-0.2049	3.52E-02	5.81E-01
	f_Actinomycetaceae	-0.2006	3.92E-02	6.04E-01
	f_Peptostreptococcaceae	-0.1988	4.10E-02	6.04E-01
	g_Turicibacter	-0.3635	1.28E-04	8.99E-02
	g_Sellimonas	-0.2884	2.71E-03	2.98E-01
	g_Phascolarctobacterium	0.2867	2.89E-03	2.98E-01
	g_Bilophila	0.2473	1.06E-02	4.85E-01
	g_Desulfovibrio	0.2412	1.28E-02	5.43E-01
	g_Actinomyces	-0.2128	2.85E-02	7.07E-01
	g_Papillibacter	0.2125	2.87E-02	7.07E-01
	g_Lachnospiraceae_UCG-010	0.2095	3.12E-02	7.30E-01
	g_Anaerofustis	-0.2049	3.52E-02	7.46E-01
	g_Pygmaiobacter	-0.2039	3.61E-02	7.46E-01
	g_Parascardovia	0.202	3.79E-02	7.46E-01
	g_Erysipelatoclostridium	-0.2007	3.92E-02	7.46E-01
Ile	f_Acidaminococcaceae	0.3536	2.00E-04	2.48E-02
	f_Muribaculaceae	0.2839	3.18E-03	1.31E-01
	f_Erysipelotrichaceae	-0.2715	4.87E-03	1.72E-01
	f_Desulfovibrionaceae	0.2511	9.41E-03	2.74E-01
	f_Streptococcaceae	-0.2266	1.95E-02	4.70E-01
	f_Actinomycetaceae	-0.2211	2.28E-02	4.87E-01
	f_Rikenellaceae	0.2004	3.94E-02	6.04E-01
	g_Phascolarctobacterium	0.3418	3.35E-04	1.57E-01
	g_Sellimonas	-0.3077	1.33E-03	2.30E-01
	g_Peptococcus	0.2739	4.50E-03	3.95E-01
	g_Erysipelatoclostridium	-0.2548	8.39E-03	4.66E-01
	g_Moryella	0.2488	1.01E-02	4.85E-01
	g_Tyzzerella	0.247	1.07E-02	4.85E-01
	g_Lachnospiraceae_UCG-010	0.2417	1.26E-02	5.43E-01
	g_Eubacterium_xylanophilum_group	0.2397	1.33E-02	5.51E-01
	g_Actinomyces	-0.2367	1.46E-02	5.69E-01
	g_Turicibacter	-0.2322	1.66E-02	5.75E-01
	g_Streptococcus	-0.2305	1.74E-02	5.75E-01
	g_Eubacterium_brachy_group	-0.2302	1.76E-02	5.75E-01

Amino acids	Bacterial Taxa	R	*P* value	FDR-*P* value
Ile	g_Desulfovibrio	0.2178	2.49E-02	6.83E-01
	g_Fenollaria	0.2166	2.57E-02	6.83E-01
	g_Pygmaiobacter	-0.212	2.92E-02	7.07E-01
	g_Alistipes	0.2004	3.94E-02	7.46E-01
	g_Eggerthella	-0.2003	3.96E-02	7.46E-01
	g_Oxalobacter	0.197	4.29E-02	7.46E-01
Val	f_Erysipelotrichaceae	-0.2872	2.83E-03	1.31E-01
	f_Desulfovibrionaceae	0.2851	3.05E-03	1.31E-01
	f_Acidaminococcaceae	0.274	4.47E-03	1.70E-01
	f_Actinomycetaceae	-0.2168	2.56E-02	4.87E-01
	f_Morganellaceae	0.2155	2.65E-02	4.87E-01
	f_Muribaculaceae	0.2139	2.77E-02	4.90E-01
	g_Peptococcus	0.2656	5.93E-03	4.24E-01
	g_Turicibacter	-0.2627	6.51E-03	4.24E-01
	g_Bilophila	0.2614	6.80E-03	4.24E-01
	g_Phascolarctobacterium	0.261	6.89E-03	4.24E-01
	g_Sellimonas	-0.2545	8.47E-03	4.66E-01
	g_Actinomyces	-0.2352	1.52E-02	5.73E-01
	g_Eggerthella	-0.2336	1.59E-02	5.74E-01
	g_Moryella	0.2179	2.48E-02	6.83E-01
	g_Desulfovibrio	0.1975	4.25E-02	7.46E-01
Phe	f_Acidaminococcaceae	0.3968	2.54E-05	1.26E-02
	f_Desulfovibrionaceae	0.3564	1.77E-04	2.48E-02
	f_Erysipelotrichaceae	-0.3417	3.37E-04	3.34E-02
	f_Rikenellaceae	0.3023	1.63E-03	1.16E-01
	f_Streptococcaceae	-0.2526	8.99E-03	2.74E-01
	f_Muribaculaceae	0.2512	9.38E-03	2.74E-01
	f_Actinomycetaceae	-0.2354	1.51E-02	4.16E-01
	f_Marinifilaceae	0.233	1.63E-02	4.23E-01
	f_Micrococcaceae	-0.2258	1.99E-02	4.70E-01
	f_Bifidobacteriaceae	-0.2203	2.32E-02	4.87E-01
	f_Anaerofustaceae	-0.218	2.48E-02	4.87E-01
	f_Veillonellaceae	-0.2167	2.56E-02	4.87E-01
	f_Oxalobacteraceae	0.2048	3.52E-02	5.81E-01
	f_Lactobacillaceae	-0.1953	4.48E-02	6.33E-01
	g_Phascolarctobacterium	0.3712	8.95E-05	8.99E-02
	g_Desulfovibrio	0.3238	7.09E-04	1.99E-01
	g_Lachnospiraceae_UCG-010	0.3125	1.11E-03	2.30E-01
	g_Erysipelatoclostridium	-0.3071	1.36E-03	2.30E-01

Amino acids	Bacterial Taxa	R	*P* value	FDR-*P* value
Phe	g_Sellimonas	-0.3032	1.58E-03	2.30E-01
	g_Alistipes	0.3023	1.63E-03	2.30E-01
	g_Turicibacter	-0.2699	5.15E-03	4.24E-01
	g_Eubacterium_xylanophilum_group	0.2613	6.83E-03	4.24E-01
	g_Bilophila	0.2608	6.93E-03	4.24E-01
	g_Dialister	-0.2539	8.62E-03	4.66E-01
	g_Streptococcus	-0.2513	9.35E-03	9.40E-01
	g_Barnesiella	0.2375	1.42E-02	5.69E-01
	g_Odoribacter	0.2316	1.69E-02	5.75E-01
	g_Rothia	-0.2258	1.99E-02	6.37E-01
	g_Bifidobacterium	-0.2204	2.32E-02	6.83E-01
	g_Actinomyces	-0.22	2.35E-02	6.83E-01
	g_Anaerofustis	-0.218	2.48E-02	6.83E-01
	g_Eubacterium_brachy_group	-0.2067	3.35E-02	7.46E-01
	g_Oxalobacter	0.2061	3.40E-02	7.46E-01
	g_Parascardovia	0.2017	3.81E-02	7.46E-01
	g_Veillonella	-0.1981	4.18E-02	7.46E-01
	g_Eubacterium_siraeum_group	0.1951	4.51E-02	7.46E-01
	g_Lactobacillus	-0.1934	4.70E-02	7.46E-01
Tyr	g_Turicibacter	-0.2346	1.55E-02	4.24E-01
	g_Eubacterium_xylanophilum_group	0.2069	3.34E-02	4.24E-01
	g_Moryella	0.2049	3.51E-02	7.46E-01
	g_Fenollaria	0.1976	4.23E-02	8.40E-01

Spearman's rank correlation was performed to evaluate the correlations of plasma amino acids with the gut microbiota.

Abbreviations: BCAAs, branched-chain amino acids; AAAs, aromatic amino acids; Leu, leucine; Ile, isoleucine; Val, valine; Phe, phenylalanine; Tyr, tyrosine; R, Spearman R value; FDR-*P*, Benjamini–Hochberg false discovery rate corrected *P* value.

Supplementary Table 4. Multivariate linear regression for bacterial taxa found to be altered in abundance between early and advanced PD

Bacterial taxa	Factor	Beta value	t_stat	*P* value
p_Desulfobacterota	Group (Early PD VS Advanced PD)	-0.194	-2.019	0.046
	Sex (Male VS Female)	-0.116	-1.197	0.234
	Age	-0.084	-0.860	0.392
	BMI	0.039	0.402	0.689
	Levodopa (use or no use)	-0.055	-0.549	0.584
	Levodopa daily dose	0.017	0.166	0.869
	LEDD	0.003	0.027	0.978
c_Desulfovibrionia	Group (Early PD VS Advanced PD)	-0.194	-2.019	0.046
	Sex (Male VS Female)	-0.116	-1.197	0.234
	Age	-0.084	-0.860	0.392
	BMI	0.039	0.402	0.689
	Levodopa (use or no use)	-0.055	-0.549	0.584
	Levodopa daily dose	0.017	0.166	0.869
	LEDD	0.003	0.027	0.978
o_Burkholderiales	Group (Early PD VS Advanced PD)	-0.223	-2.113	0.037
	Sex (Male VS Female)	-0.087	-0.873	0.385
	Age	0.040	0.383	0.703
	BMI	0.152	1.428	0.156
	Levodopa (use or no use)	-0.101	-0.861	0.391
	Levodopa daily dose	-0.241	-1.025	0.308
	LEDD	0.236	1.086	0.280
o_Desulfovibrionales	Group (Early PD VS Advanced PD)	-0.194	-2.019	0.046
	Sex (Male VS Female)	-0.116	-1.197	0.234
	Age	-0.084	-0.860	0.392
	BMI	0.039	0.402	0.689
	Levodopa (use or no use)	-0.055	-0.549	0.584
	Levodopa daily dose	0.017	0.166	0.869
	LEDD	0.003	0.027	0.978
o_Lachnospirales	Group (Early PD VS Advanced PD)	-0.282	-2.998	0.003
	Sex (Male VS Female)	0.107	1.132	0.260
	Age	0.089	0.937	0.351
	BMI	0.047	0.500	0.618
	Levodopa (use or no use)	0.044	0.449	0.654
	Levodopa daily dose	0.026	0.263	0.793

	LEDD	0.011	0.116	0.908
f_Butyricicoccaceae	Group (Early PD VS Advanced PD)	-0.020	-0.185	0.853
	Sex (Male VS Female)	-0.024	-0.231	0.818
	Age	-0.052	-0.487	0.627
	BMI	0.100	0.905	0.368
	Levodopa (use or no use)	0.130	1.076	0.285
	Levodopa daily dose	-0.004	-0.017	0.987
	LEDD	0.023	0.104	0.917
f_Desulfovibrionaceae	Group (Early PD VS Advanced PD)	-0.194	-2.019	0.046
	Sex (Male VS Female)	-0.116	-1.197	0.234
	Age	-0.084	-0.860	0.392
	BMI	0.039	0.402	0.689
	Levodopa (use or no use)	-0.055	-0.549	0.584
	Levodopa daily dose	0.017	0.166	0.869
	LEDD	0.003	0.027	0.978
f_Lachnospiraceae	Group (Early PD VS Advanced PD)	-0.282	-2.998	0.003
	Sex (Male VS Female)	0.107	1.130	0.261
	Age	0.089	0.937	0.351
	BMI	0.047	0.496	0.621
	Levodopa (use or no use)	0.044	0.452	0.652
	Levodopa daily dose	0.026	0.264	0.792
	LEDD	0.011	0.115	0.908
f_Leuconostocaceae	Group (Early PD VS Advanced PD)	0.107	1.001	0.319
	Sex (Male VS Female)	-0.154	-1.525	0.130
	Age	-0.015	-0.142	0.888
	BMI	-0.059	-0.542	0.589
	Levodopa (use or no use)	-0.007	-0.055	0.956
	Levodopa daily dose	-0.163	-0.682	0.497
	LEDD	0.272	1.236	0.219
f_Sutterellaceae	Group (Early PD VS Advanced PD)	-0.116	-1.089	0.279
	Sex (Male VS Female)	-0.125	-1.240	0.218
	Age	-0.088	-0.842	0.402
	BMI	-0.176	-1.635	0.105
	Levodopa (use or no use)	-0.032	-0.271	0.787
	Levodopa daily dose	0.095	0.403	0.688
	LEDD	-0.112	-0.514	0.608

f_unclassified_o__ Coriobacteriales	Group (Early PD VS Advanced PD)	0.198	2.064	0.042
	Sex (Male VS Female)	-0.098	-1.015	0.312
	Age	-0.032	-0.329	0.743
	BMI	-0.095	-0.989	0.325
	Levodopa (use or no use)	-0.015	-0.145	0.885
	Levodopa daily dose	0.070	0.699	0.486
	LEDD	0.039	0.390	0.697
g_Butyricicoccus	Group (Early PD VS Advanced PD)	-0.025	-0.231	0.817
	Sex (Male VS Female)	-0.017	-0.165	0.869
	Age	-0.045	-0.421	0.675
	BMI	0.102	0.927	0.356
	Levodopa (use or no use)	0.121	1.001	0.319
	Levodopa daily dose	-0.014	-0.056	0.956
	LEDD	0.029	0.131	0.896
g_Lachnospiraceae_ UCG-010	Group (Early PD VS Advanced PD)	-0.191	-1.983	0.050
	Sex (Male VS Female)	-0.141	-1.470	0.145
	Age	-0.018	-0.188	0.851
	BMI	0.016	0.169	0.867
	Levodopa (use or no use)	-0.010	-0.097	0.923
	Levodopa daily dose	-0.034	-0.339	0.735
	LEDD	0.030	0.301	0.764
g_Parasutterella	Group (Early PD VS Advanced PD)	-0.338	-3.658	< 0.001
	Sex (Male VS Female)	0.032	0.339	0.735
	Age	-0.009	-0.099	0.922
	BMI	-0.071	-0.764	0.447
	Levodopa (use or no use)	0.149	1.568	0.120
	Levodopa daily dose	-0.084	-0.880	0.381
	LEDD	-0.077	-0.809	0.420
g_Ruminococcus_ gnavus_group	Group (Early PD VS Advanced PD)	-0.064	-0.595	0.553
	Sex (Male VS Female)	0.106	1.033	0.304
	Age	-0.119	-1.122	0.264
	BMI	0.012	0.113	0.910
	Levodopa (use or no use)	-0.002	-0.019	0.985
	Levodopa daily dose	0.055	0.228	0.820
	LEDD	-0.126	-0.566	0.573

g_Subdoligranulum	Group (Early PD VS Advanced PD)	0.313	3.365	0.001
	Sex (Male VS Female)	0.092	0.977	0.331
	Age	0.136	1.453	0.149
	BMI	0.064	0.691	0.491
	Levodopa (use or no use)	0.089	0.918	0.361
	Levodopa daily dose	-0.030	-0.313	0.755
	LEDD	-0.023	-0.243	0.809
g_unclassified_o__ Coriobacteriales	Group (Early PD VS Advanced PD)	0.198	2.064	0.042
	Sex (Male VS Female)	-0.098	-1.015	0.312
	Age	-0.032	-0.329	0.743
	BMI	-0.095	-0.989	0.325
	Levodopa (use or no use)	-0.015	-0.145	0.885
	Levodopa daily dose	0.070	0.699	0.486
	LEDD	0.039	0.390	0.697
g_Weissella	Group (Early PD VS Advanced PD)	0.107	1.004	0.318
	Sex (Male VS Female)	-0.155	-1.529	0.129
	Age	-0.015	-0.141	0.888
	BMI	-0.057	-0.531	0.597
	Levodopa (use or no use)	-0.006	-0.047	0.963
	Levodopa daily dose	-0.158	-0.664	0.508
	LEDD	0.269	1.222	0.225

Beta value represents the regression coefficient.

The alteration in abundance of bacterial taxa between early and advanced PD patients was further evaluated using multivariate linear regression, adjusting for age, sex, BMI, levodopa (use or no use), levodopa daily dose, and LEDD.

Abbreviations: BMI, body mass index; LEDD, levodopa equivalent daily dose.

Supplementary Table 5. Correlations between the altered microbial taxa and plasma BCAAs and AAAs in patients with early and advanced PD

**Amino acids**	**Bacteria taxa**	**R**	***P* value**	**FDR-*P* value**
Early PD				
Leu	g_Subdoligranulum ↑	0.3855	6.81E-03	9.95E-02
Ile	g_Subdoligranulum ↑	0.3752	8.60E-03	9.95E-02
Tyr	g_Subdoligranulum ↑	0.2967	4.06E-02	3.25E-01
Phe	g_Subdoligranulum ↑	0.2977	3.98E-02	3.25E-01
	f_Desulfovibrionaceae ↓	0.5000	2.97E-04	5.93E-03
	o_Desulfovibrionales ↓	0.5000	2.97E-04	5.93E-03
	c_Desulfovibrionia ↓	0.5000	2.97E-04	5.93E-03
	p_Desulfobacterota ↓	0.5000	2.97E-04	5.93E-03
Advanced PD				
Leu	f_Desulfovibrionaceae ↓	0.3449	8.01E-03	1.38E-01
	o_Desulfovibrionales ↓	0.3449	8.01E-03	1.38E-01
	c_Desulfovibrionia ↓	0.3449	8.01E-03	1.38E-01
	p_Desulfobacterota ↓	0.3449	8.01E-03	1.38E-01
Val	f_Desulfovibrionaceae ↓	0.3269	1.23E-02	1.38E-01
	o_Desulfovibrionales ↓	0.3269	1.23E-02	1.38E-01
	c_Desulfovibrionia ↓	0.3269	1.23E-02	1.38E-01
	p_Desulfobacterota ↓	0.3269	1.23E-02	1.38E-01

Spearman's rank correlation was performed to evaluate the correlations between plasma amino acids and the gut microbiota.

↑, Significantly increased in advanced PD patients.

↓, Significantly reduced in advanced PD patients.

Abbreviations: BCAAs, branched-chain amino acids; AAAs, aromatic amino acids; Leu, leucine; Ile, isoleucine; Val, valine; Phe, phenylalanine; Tyr, tyrosine; R, Spearman R value; FDR-*P*, Benjamini–Hochberg false discovery rate corrected *P* value.

Supplementary Table 6. Multivariate linear regression for the correlations between the altered microbial taxa and plasma Phe adjusting for confounding factors in early PD patients

Dependent Variable	Independent Variables	Beta value	t_stat	*P* value
Phe	f_Desulfovibrionaceae	0.438	3.513	0.001
	Sex (Male VS Female)	0.093	0.720	0.475
	Age	0.127	0.917	0.365
	BMI	0.187	1.317	0.195
	Levodopa (use or no use)	0.068	0.418	0.678
	Levodopa daily dose	-0.644	-2.060	0.046
	LEDD	0.639	2.319	0.026
	o_Desulfovibrionales	0.438	3.513	0.001
	Sex (Male VS Female)	0.093	0.720	0.475
	Age	0.127	0.917	0.365
	BMI	0.187	1.317	0.195
	Levodopa (use or no use)	0.068	0.418	0.678
	Levodopa daily dose	-0.644	-2.060	0.046
	LEDD	0.639	2.319	0.026
	c_Desulfovibrionia	0.438	3.513	0.001
	Sex (Male VS Female)	0.093	0.720	0.475
	Age	0.127	0.917	0.365
	BMI	0.187	1.317	0.195
	Levodopa (use or no use)	0.068	0.418	0.678
	Levodopa daily dose	-0.644	-2.060	0.046
	LEDD	0.639	2.319	0.026
	p_Desulfobacterota	0.438	3.513	0.001
	Sex (Male VS Female)	0.093	0.720	0.475
	Age	0.127	0.917	0.365
	BMI	0.187	1.317	0.195
	Levodopa (use or no use)	0.068	0.418	0.678
	Levodopa daily dose	-0.644	-2.060	0.046
	LEDD	0.639	2.319	0.026

Beta value represents the regression coefficient.

The correlations between the altered microbial taxa and plasma Phe in patients with early PD were further evaluated using multivariate linear regression, adjusting for age, sex, BMI, levodopa (use or no use), levodopa daily dose, and LEDD.

Abbreviations: Phe, phenylalanine; BMI, body mass index; LEDD, levodopa equivalent daily dose.

Supplementary Table 7. Pathways with different abundances of predicted genes between early and advanced PD patients, as inferred using PICRUSt2 analysis of fecal microbiomes

MetaCyc Pathway	Advanced PD Mean proportion (%)	Early PD Mean proportion (%)	*P* Value	FDR-*P* Value
TCA cycle IV (2-oxoglutarate decarboxylase)	0.52	0.55	9.99E-04	1.88E-02
superpathway of branched-chain amino acid biosynthesis	0.63	0.68	9.99E-04	2.06E-02
pyruvate fermentation to acetate and lactate II	0.38	0.33	9.99E-04	2.16E-02
TCA cycle V (2-oxoglutarate:ferredoxin oxidoreductase)	0.56	0.62	9.99E-04	2.28E-02
S-adenosyl-L-methionine cycle I	0.38	0.32	9.99E-04	2.40E-02
L-ornithine biosynthesis	0.46	0.51	9.99E-04	2.54E-02
superpathway of pyrimidine deoxyribonucleosides degradation	0.26	0.22	9.99E-04	2.70E-02
L-valine biosynthesis	0.65	0.71	2.00E-03	2.79E-02
L-isoleucine biosynthesis II	0.69	0.75	9.99E-04	2.88E-02
colanic acid building blocks biosynthesis	0.26	0.28	2.00E-03	2.88E-02
L-isoleucine biosynthesis I (from threonine)	0.65	0.71	2.00E-03	2.98E-02
superpathway of GDP-mannose-derived O-antigen building blocks biosynthesis	0.28	0.37	9.99E-04	3.09E-02
acetylene degradation	0.43	0.36	2.00E-03	3.09E-02
GDP-mannose biosynthesis	0.49	0.54	2.00E-03	3.20E-02
thiamin salvage II	0.42	0.46	2.00E-03	3.33E-02
superpathway of 2,3-butanediol biosynthesis	0.13	0.09	9.99E-04	3.33E-02
hexitol fermentation to lactate, formate, ethanol and acetate	0.27	0.23	9.99E-04	3.60E-02
L-tryptophan biosynthesis	0.50	0.55	3.00E-03	3.82E-02
superpathway of histidine, purine, and pyrimidine biosynthesis	0.45	0.42	9.99E-04	3.93E-02
superpathway of L-threonine metabolism	0.07	0.05	3.00E-03	3.93E-02
sucrose degradation III (sucrose invertase)	0.39	0.33	3.00E-03	4.06E-02
NAD biosynthesis I (from aspartate)	0.39	0.43	9.99E-04	4.33E-02
pyruvate fermentation to isobutanol (engineered)	0.77	0.82	4.00E-03	4.81E-02
TCA cycle I (prokaryotic)	0.59	0.64	9.99E-04	4.81E-02

L-glutamate and L-glutamine biosynthesis	0.28	0.25	4.00E-03	4.94E-02
L-isoleucine biosynthesis III	0.58	0.63	9.99E-04	5.41E-02
superpathway of L-isoleucine biosynthesis I	0.56	0.60	5.00E-03	5.41E-02
flavin biosynthesis I (bacteria and plants)	0.46	0.49	5.00E-03	5.55E-02
polyisoprenoid biosynthesis (E. coli)	0.46	0.49	5.00E-03	5.69E-02
purine ribonucleosides degradation	0.32	0.27	5.00E-03	5.85E-02
mixed acid fermentation	0.42	0.39	5.99E-03	6.04E-02
peptidoglycan biosynthesis V	0.10	0.06	9.99E-04	6.18E-02
adenosine deoxyribonucleotides de novo biosynthesis II	0.58	0.61	6.99E-03	6.44E-02
guanosine deoxyribonucleotides de novo biosynthesis II	0.58	0.61	6.99E-03	6.58E-02
superpathway of purine deoxyribonucleosides degradation	0.31	0.28	6.99E-03	6.73E-02
anhydromuropeptides recycling	0.53	0.49	6.99E-03	6.88E-02
methylerythritol phosphate pathway I	0.43	0.46	7.99E-03	7.06E-02
methylerythritol phosphate pathway II	0.43	0.46	7.99E-03	7.21E-02
flavin biosynthesis II (archaea)	0.03	0.02	8.99E-03	7.63E-02
heme biosynthesis II (anaerobic)	0.47	0.51	8.99E-03	7.79E-02
superpathway of L-serine and glycine biosynthesis I	0.55	0.58	9.99E-03	8.32E-02
L-histidine degradation II	0.09	0.07	1.10E-02	8.81E-02
superpathway of L-aspartate and L-asparagine biosynthesis	0.31	0.28	1.10E-02	8.98E-02
chorismate biosynthesis I	0.55	0.57	1.30E-02	1.00E-01
superpathway of aromatic amino acid biosynthesis	0.58	0.60	1.30E-02	1.02E-01
arginine, ornithine and proline interconversion	0.15	0.12	1.50E-02	1.06E-01
L-lysine biosynthesis II	0.23	0.18	1.70E-02	1.10E-01
tetrapyrrole biosynthesis II (from glycine)	0.46	0.50	1.60E-02	1.10E-01
superpathway of polyamine biosynthesis I	0.25	0.22	1.50E-02	1.10E-01
chorismate biosynthesis from 3-dehydroquinate	0.54	0.56	1.70E-02	1.11E-01
L-arginine biosynthesis IV (archaebacteria)	0.46	0.50	1.80E-02	1.13E-01
tetrapyrrole biosynthesis I (from glutamate)	0.46	0.49	1.70E-02	1.13E-01
superpathway of guanosine nucleotides de novo biosynthesis II	0.56	0.58	1.90E-02	1.14E-01

superpathway of geranylgeranyl diphosphate biosynthesis II (via MEP)	0.44	0.47	1.80E-02	1.15E-01
superpathway of adenosine nucleotides de novo biosynthesis II	0.58	0.61	1.70E-02	1.15E-01
superpathway of L-threonine biosynthesis	0.53	0.56	2.00E-02	1.15E-01
pyrimidine deoxyribonucleotides de novo biosynthesis II	0.37	0.35	1.90E-02	1.16E-01
Calvin-Benson-Bassham cycle	0.60	0.62	2.10E-02	1.16E-01
CMP-3-deoxy-D-manno-octulosonate biosynthesis I	0.31	0.35	2.00E-02	1.17E-01
L-arginine biosynthesis I (via L-ornithine)	0.47	0.50	1.90E-02	1.17E-01
coenzyme A biosynthesis I	0.48	0.51	2.10E-02	1.18E-01
Kdo transfer to lipid IVA III (Chlamydia)	0.29	0.33	2.00E-02	1.19E-01
superpathway of pyrimidine ribonucleosides salvage	0.43	0.41	2.10E-02	1.20E-01
3-hydroxypropanoate/4-hydroxybutanate cycle	0.01	0.00	2.20E-02	1.20E-01
biotin biosynthesis II	0.02	0.01	2.30E-02	1.23E-01
superpathway of arginine and polyamine biosynthesis	0.32	0.30	2.50E-02	1.24E-01
pyrimidine deoxyribonucleotide phosphorylation	0.47	0.50	2.30E-02	1.24E-01
pyruvate fermentation to propanoate I	0.32	0.38	2.40E-02	1.25E-01
CDP-diacylglycerol biosynthesis I	0.60	0.63	2.50E-02	1.26E-01
superpathway of adenosine nucleotides de novo biosynthesis I	0.61	0.64	2.50E-02	1.27E-01
CDP-diacylglycerol biosynthesis II	0.60	0.63	2.50E-02	1.29E-01
superpathway of guanosine nucleotides de novo biosynthesis I	0.58	0.61	3.00E-02	1.37E-01
superpathway of purine nucleotides de novo biosynthesis I	0.55	0.58	3.10E-02	1.37E-01
lipid IVA biosynthesis	0.29	0.33	2.80E-02	1.38E-01
adenosine nucleotides degradation IV	0.01	0.00	2.90E-02	1.38E-01
superpathway of pyrimidine ribonucleotides de novo biosynthesis	0.56	0.59	3.00E-02	1.38E-01
L-methionine salvage cycle III	0.09	0.07	3.10E-02	1.38E-01
superpathway of (R,R)-butanediol biosynthesis	0.12	0.09	3.20E-02	1.38E-01
superpathway of purine nucleotides de novo biosynthesis II	0.48	0.46	2.90E-02	1.39E-01
S-methyl-5-thio-α-D-ribose 1-phosphate degradation	0.08	0.05	3.00E-02	1.40E-01

superpathway of sulfur oxidation (Acidianus ambivalens)	0.13	0.10	3.10E-02	1.40E-01
L-arginine biosynthesis II (acetyl cycle)	0.51	0.54	3.20E-02	1.40E-01
pyrimidine deoxyribonucleosides salvage	0.31	0.28	3.00E-02	1.41E-01
mevalonate pathway II (archaea)	0.02	0.01	3.30E-02	1.41E-01
pentose phosphate pathway (non-oxidative branch)	0.72	0.76	3.50E-02	1.47E-01
pantothenate and coenzyme A biosynthesis I	0.46	0.49	3.50E-02	1.48E-01
5-aminoimidazole ribonucleotide biosynthesis I	0.56	0.59	3.60E-02	1.50E-01
superpathway of glycolysis and Entner-Doudoroff	0.46	0.45	3.80E-02	1.52E-01
enterobacterial common antigen biosynthesis	0.12	0.10	3.70E-02	1.52E-01
5-aminoimidazole ribonucleotide biosynthesis II	0.55	0.58	3.80E-02	1.54E-01
superpathway of phospholipid biosynthesis I (bacteria)	0.58	0.60	4.00E-02	1.54E-01
guanosine ribonucleotides de novo biosynthesis	0.56	0.59	3.90E-02	1.55E-01
superpathway of 5-aminoimidazole ribonucleotide biosynthesis	0.55	0.58	3.80E-02	1.55E-01
dTDP-N-acetylthomosamine biosynthesis	0.16	0.14	4.10E-02	1.56E-01
ectoine biosynthesis	0.13	0.10	4.00E-02	1.56E-01
teichoic acid (poly-glycerol) biosynthesis	0.05	0.04	4.10E-02	1.57E-01
peptidoglycan maturation (meso-diaminopimelate containing)	0.56	0.52	4.00E-02	1.57E-01
nicotinate degradation I	0.04	0.02	4.30E-02	1.62E-01
inosine-5'-phosphate biosynthesis I	0.48	0.51	4.60E-02	1.66E-01
O-antigen building blocks biosynthesis (E. coli)	0.44	0.47	4.70E-02	1.67E-01
adenosine ribonucleotides de novo biosynthesis	0.59	0.62	4.60E-02	1.67E-01
thiazole biosynthesis II (Bacillus)	0.17	0.15	4.70E-02	1.68E-01
superpathway of glycerol degradation to 1,3-propanediol	0.02	0.01	4.60E-02	1.69E-01
glycolysis III (from glucose)	0.57	0.59	4.60E-02	1.70E-01
UMP biosynthesis	0.57	0.59	4.90E-02	1.71E-01
dTDP-L-rhamnose biosynthesis I	0.47	0.50	4.90E-02	1.72E-01

Predicted functional microbiota profiling was performed using PICRUSt2. The metabolic pathways were annotated by the MetaCyc database. Differences between groups were identified using White's non-parametric t-test with FDR correction using the STAMP software.

Abbreviations: PICRUSt2, Phylogenetic Investigation of Communities by Reconstruction of Unobserved States 2; FDR-*P*, Benjamini–Hochberg false discovery rate corrected *P* value; STAMP, Statistical Analysis of Metagenomic Profiles.

Supplementary Table 8. HPLC-FLD validation parameters of analytical procedure for plasma BCAAs and AAAs

	Linearity		Precision (RSD[a], %)		Accuracy (% accuracy[b])		LODs (μg/mL)	LOQs (μg/mL)
	Concentration range (μg/mL)	R^2	Interday assay	Intraday assay	Interday assay	Intraday assay		
Leu	0.66–5.26	0.9872	5.53	8.99	94.68	92.54	0.10	0.50
Ile	0.23–3.64	0.9850	3.63	3.95	96.58	97.65	0.02	0.05
Val	0.39–6.19	0.9833	4.94	1.28	98.48	101.55	0.05	0.10
Phe	0.22–3.51	0.9717	8.95	8.10	95.07	101.05	0.05	0.10
Tyr	1.41–22.60	0.9983	8.46	1.77	102.25	99.73	0.05	0.10

[a]: Average RSD obtained from six replicates.

[b]: Average % accuracy obtained from six replicates.

Abbreviations: HPLC-FLD, high-performance liquid chromatography with fluorescence detection; BCAAs, branched-chain amino acids; AAAs, aromatic amino acids; Leu, leucine; Ile, isoleucine; Val, valine; Phe, phenylalanine; Tyr, tyrosine; RSD, relative standard deviation; LODs, limits of detection; LOQs, limits of quantification.

Supplementary Table 9. Primers for quantitative real-time PCR

Targeting gene	Primers	Sequence 5'-3'
ilvB	Forward	CGGACTNGGCHCNATGGG
	Reverse	ACATTGYCTGCCARTGBYG
ilvC	Forward	GATCGGCTAYGGCGBVCAGGGWC
	Reverse	CCCGGACCTTTVGGNGCVAYCAT
ilvD	Forward	CGAAGGGYGGHCCBGGNATG
	Reverse	RCCNCTGAAACGWCCRTCKGT
ilvE	Forward	GAGTGCGGCGSYTGCGGHACBGCNGC
	Reverse	GGTTCRKCACCVNACTGRATACC
ilvN	Forward	GCCGCCAARTMAAYATCGAGAG
	Reverse	GAATAACGTCDATCTTCTTYTCRATCTG

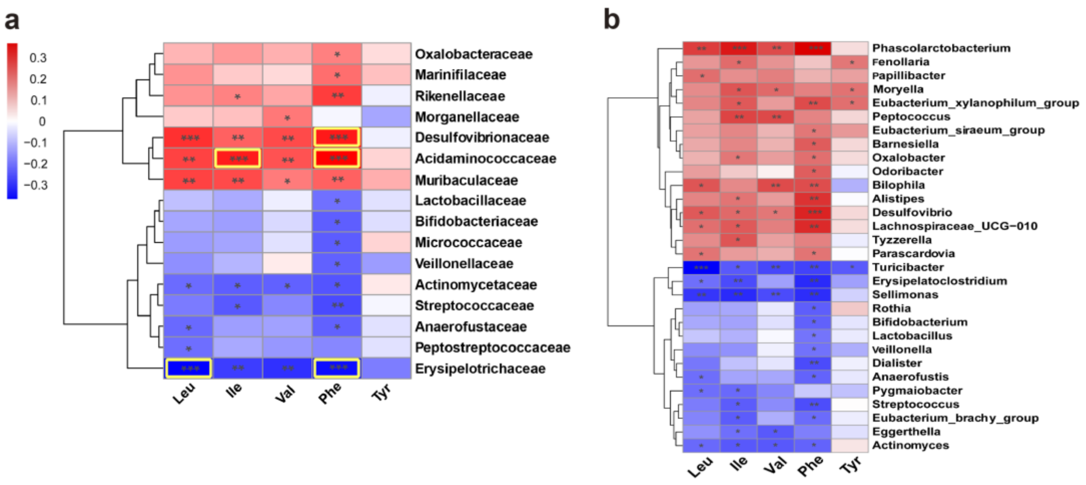

Supplementary Figure 1. Correlations between plasma BCAAs and AAAs and the gut microbiota. The heat maps represent Spearman's rank correlation of BCAAs and AAAs with the gut microbiota at the family level (a) and genus level (b). The significant correlations between gut microbiota and BCAAs and AAAs (FDR-*P* < 0.05) are highlighted (yellow border). **P* < 0.05, ***P* < 0.01, ****P* < 0.001. *Abbreviations:* BCAAs, branched-chain amino acids; AAAs, aromatic amino acids; Leu, leucine; Ile, isoleucine; Val, valine; Phe, phenylalanine; Tyr, tyrosine; FDR, Benjamini–Hochberg false discovery rate.

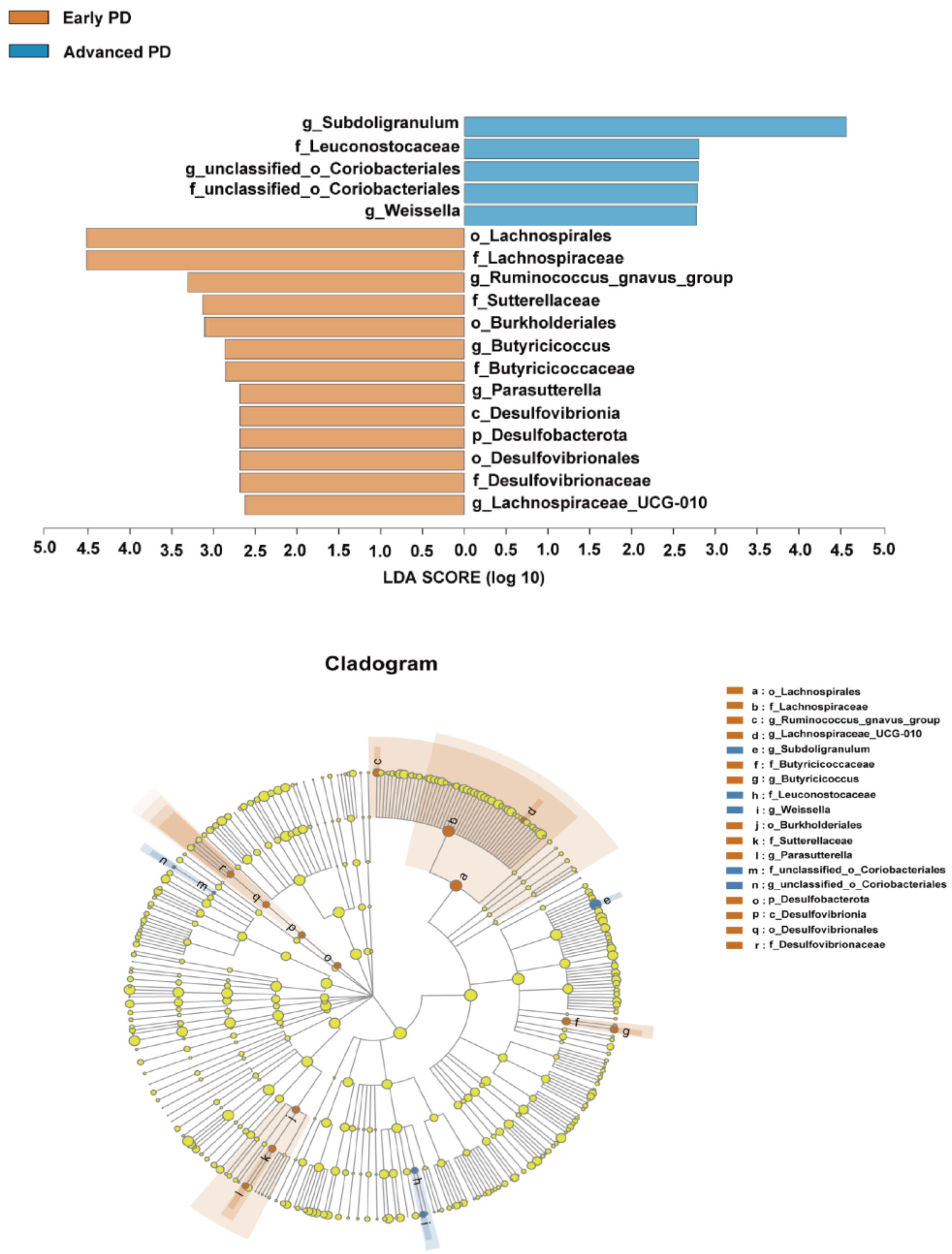

Supplementary Figure 2. The alteration of fecal microbiota between early and advanced PD patients. LEfSe analysis revealed remarkable microbial differences between early and advanced PD patients. The LDA scores (log_{10}) > 2.5 and *P* < 0.05 are listed. A cladogram using the LEfSe method indicates the phylogenetic distribution of the gut microbiota.

Abbreviations: LEfSe, linear discriminant analysis (LDA) effect size; p, phylum; c, class; o, order; f, family; g, genus.

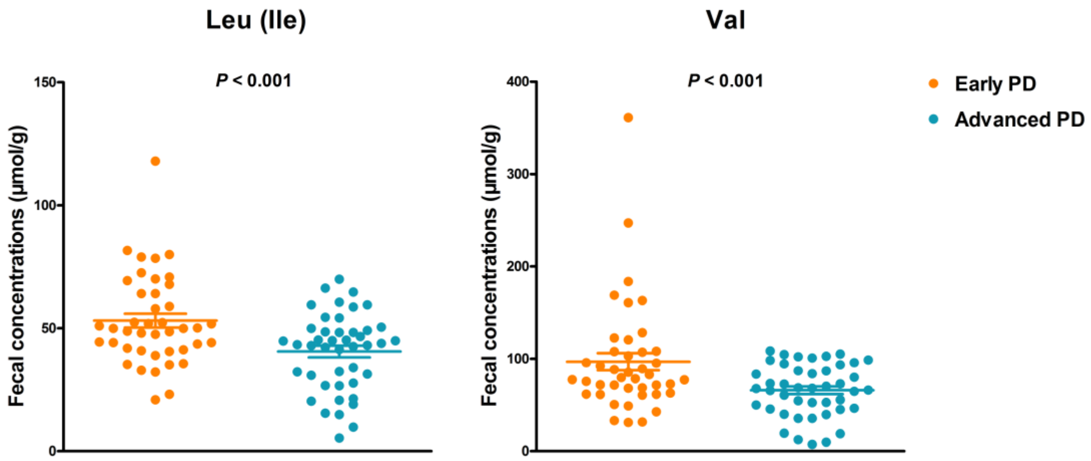

Supplementary Figure 3. Comparisons of fecal BCAA concentrations between early and advanced PD patients. Comparing with patients with early PD (n = 42), fecal BCAA concentrations were lower in advanced PD patients (n = 44). Differences between groups were assessed using ANCOVA, adjusting for age, sex, BMI, levodopa (use or no use), and LEDD. Data are presented as mean ± SEM. *Abbreviations:* Leu, leucine; Ile, isoleucine; Val, valine; BCAA, branched-chain amino acid; ANCOVA, analysis of covariance; BMI, body mass index; LEDD, levodopa equivalent daily dose; SEM, standard error of the mean.
