# Supplementary material for: Plasma branched-chain and aromatic amino acids correlate with the gut microbiota and severity of Parkinson’s disease
Source: NPJ Parkinsons Dis. 2022 Apr 21;8:48. doi: 10.1038/s41531-022-00312-z (PMC9023571; doi:10.1038/s41531-022-00312-z)
Supplement: Supplementary file 2 — Reporting Summary [file 41531_2022_312_MOESM2_ESM.pdf]

nature portfolio

Corresponding author(s): Qin Xiao, Xiaodong Yang

Last updated by author(s): Mar 11, 2022

Reporting Summary

Nature Portfolio wishes to improve the reproducibility of the work that we publish. This form provides structure for consistency and transparency in reporting. For further information on Nature Portfolio policies, see our Editorial Policies and the Editorial Policy Checklist.

Statistics

For all statistical analyses, confirm that the following items are present in the figure legend, table legend, main text, or Methods section.

n/a	Confirmed	
☐	☒	The exact sample size (n) for each experimental group/condition, given as a discrete number and unit of measurement
☐	☒	A statement on whether measurements were taken from distinct samples or whether the same sample was measured repeatedly
☐	☒	The statistical test(s) used AND whether they are one- or two-sided<br>*Only common tests should be described solely by name; describe more complex techniques in the Methods section.*
☐	☒	A description of all covariates tested
☐	☒	A description of any assumptions or corrections, such as tests of normality and adjustment for multiple comparisons
☐	☒	A full description of the statistical parameters including central tendency (e.g. means) or other basic estimates (e.g. regression coefficient) AND variation (e.g. standard deviation) or associated estimates of uncertainty (e.g. confidence intervals)
☐	☒	For null hypothesis testing, the test statistic (e.g. F, t, r) with confidence intervals, effect sizes, degrees of freedom and P value noted<br>*Give P values as exact values whenever suitable.*
☒	☐	For Bayesian analysis, information on the choice of priors and Markov chain Monte Carlo settings
☒	☐	For hierarchical and complex designs, identification of the appropriate level for tests and full reporting of outcomes
☒	☐	Estimates of effect sizes (e.g. Cohen's d, Pearson's r), indicating how they were calculated

Our web collection on statistics for biologists contains articles on many of the points above.

Software and code

Policy information about availability of computer code

Data collection	no software was used
Data analysis	QIIME2 (version 2020.2); SILVA database (version 138); mothur (version 1.35.1); R software (version 4.0.3; R Foundation for Statistical Computing, Vienna, Austria); PICRUSt2 (version 2.2.0-b); MetaCyc database (version 23.1); STAMP software (version 2.1.3); SPSS software (version 22.0, IBM Corporation, Armonk, NY, USA).

For manuscripts utilizing custom algorithms or software that are central to the research but not yet described in published literature, software must be made available to editors and reviewers. We strongly encourage code deposition in a community repository (e.g. GitHub). See the Nature Portfolio guidelines for submitting code & software for further information.

Data

Policy information about availability of data

All manuscripts must include a data availability statement. This statement should provide the following information, where applicable:

- Accession codes, unique identifiers, or web links for publicly available datasets
- A description of any restrictions on data availability
- For clinical datasets or third party data, please ensure that the statement adheres to our policy

Sequences generated and analyzed during this study are accessible from the National Center for Biotechnology Information (NCBI) Sequence Read Archive (SRA) under the accession code SRP337726. The key data are included in this published article and its supplementary information files. Other datasets are available from the corresponding author upon reasonable request.

Field-specific reporting

Please select the one below that is the best fit for your research. If you are not sure, read the appropriate sections before making your selection.

☒ Life sciences ☐ Behavioural & social sciences ☐ Ecological, evolutionary & environmental sciences

For a reference copy of the document with all sections, see nature.com/documents/nr-reporting-summary-flat.pdf

Life sciences study design

All studies must disclose on these points even when the disclosure is negative.

Sample size	We have included 106 PD patients and 114 controls. This was the maximum number of participants fullfilling the inclusion criteria, and after applying the exclusion criteria.
Data exclusions	No samples were excluded.
Replication	Findings were routinely checked and confirmed by two different investigators throughout the analyses.
Randomization	n/a
Blinding	Our study aimed to to explore plasma BCAA and AAA profiles in PD patients and the participants were grouped according to their own disease status.

Reporting for specific materials, systems and methods

We require information from authors about some types of materials, experimental systems and methods used in many studies. Here, indicate whether each material, system or method listed is relevant to your study. If you are not sure if a list item applies to your research, read the appropriate section before selecting a response.

Materials & experimental systems

n/a	Involved in the study	
☒	☐	Antibodies
☒	☐	Eukaryotic cell lines
☒	☐	Palaeontology and archaeology
☒	☐	Animals and other organisms
☐	☒	Human research participants
☒	☐	Clinical data
☒	☐	Dual use research of concern

Methods

n/a	Involved in the study	
☒	☐	ChIP-seq
☒	☐	Flow cytometry
☒	☐	MRI-based neuroimaging

Human research participants

Policy information about studies involving human research participants

Population characteristics	Demographics and clinical characteristics of study participants are detailed in the manuscript.
Recruitment	Parkinson's disease patients were recruited from the Movement Disorders Clinic at the Department of Neurology and Institute of Neurology, Ruijin Hospital. Controls matched by age, sex, and nutritional status were recruited over the same period.
Ethics oversight	This study was approved by the Research Ethics Committee, Ruijin Hospital, Shanghai Jiao Tong University School of Medicine, Shanghai, China.

Note that full information on the approval of the study protocol must also be provided in the manuscript.
